# Multiplexed MRM-based proteomics for identification of circulating proteins as biomarkers of cardiovascular damage progression associated with diabetes mellitus

**DOI:** 10.1186/s12933-024-02125-1

**Published:** 2024-01-20

**Authors:** Francesco Piarulli, Cristina Banfi, Eugenio Ragazzi, Erica Gianazza, Marco Munno, Massimo Carollo, Pietro Traldi, Annunziata Lapolla, Giovanni Sartore

**Affiliations:** 1 Department of Medicine – DIMED, University of Padova, Padova, Italy; 2 Centro Cardiologico Monzino, IRCCS, Milano, 20138 Italy; 3 Department of Pharmaceutical and Pharmacological Sciences, University of Padova, Padova, Italy; 4 Istituto di Ricerca Pediatrica Città della Speranza, Padova, Italy

**Keywords:** Proteomics, Blood proteins, Diabetes mellitus, Cardiovascular disease, Mass spectrometry, Multiple reaction monitoring, Gene Ontology analysis

## Abstract

**Background:**

Type 2 diabetes mellitus (T2DM) increases the risk of coronary heart disease (CHD) by 2–4 fold, and is associated with endothelial dysfunction, dyslipidaemia, insulin resistance, and chronic hyperglycaemia. The aim of this investigation was to assess, by a multimarker mass spectrometry approach, the predictive role of circulating proteins as biomarkers of cardiovascular damage progression associated with diabetes mellitus.

**Methods:**

The study considered 34 patients with both T2DM and CHD, 31 patients with T2DM and without CHD, and 30 patients without diabetes with a diagnosis of CHD. Plasma samples of subjects were analysed through a multiplexed targeted liquid chromatography mass spectrometry (LC-MS)-based assay, namely Multiple Reaction Monitoring (MRM), allowing the simultaneous detection of peptides derived from a protein of interest. Gene Ontology (GO) Analysis was employed to identify enriched GO terms in the biological process, molecular function, or cellular component categories. Non-parametric multivariate methods were used to classify samples from patients and evaluate the relevance of the analysed proteins’ panel.

**Results:**

A total of 81 proteins were successfully quantified in the human plasma samples. Gene Ontology analysis assessed terms related to blood microparticles, extracellular exosomes and collagen-containing extracellular matrix. Preliminary evaluation using analysis of variance (ANOVA) of the differences in the proteomic profile among patient groups identified 13 out of the 81 proteins as significantly different. Multivariate analysis, including cluster analysis and principal component analysis, identified relevant grouping of the 13 proteins. The first main cluster comprises apolipoprotein C-III, apolipoprotein C-II, apolipoprotein A-IV, retinol-binding protein 4, lysozyme C and cystatin-C; the second one includes, albeit with sub-grouping, alpha 2 macroglobulin, afamin, kininogen 1, vitronectin, vitamin K-dependent protein S, complement factor B and mannan-binding lectin serine protease 2. Receiver operating characteristic (ROC) curves obtained with the 13 selected proteins using a nominal logistic regression indicated a significant overall distinction (*p* < 0.001) among the three groups of subjects, with area under the ROC curve (AUC) ranging 0.91–0.97, and sensitivity and specificity ranging from 85 to 100%.

**Conclusions:**

Targeted mass spectrometry approach indicated 13 multiple circulating proteins as possible biomarkers of cardiovascular damage progression associated with T2DM, with excellent classification results in terms of sensitivity and specificity.

**Supplementary Information:**

The online version contains supplementary material available at 10.1186/s12933-024-02125-1.

## Introduction

Diabetes mellitus (DM), mainly type 2 diabetes mellitus (T2DM, which accounts for 90–95% of patients), is associated with a 2–4-fold increase in the risk of coronary heart disease (CHD), that is the principal cause of morbidity and mortality in developed countries [1]. DM is a predictor of poor prognosis following acute myocardial infarction (MI), congestive heart failure, and coronary revascularisation. The increased onset of atherosclerosis and atherothrombosis in patients affected by DM has been attributed to various factors, including endothelial dysfunction, dyslipidaemia, chronic hyperglycaemia and insulin resistance. Additionally, free fatty acids and glycosylation end products [2] contribute to processes that lead to vascular damage, such as vasoconstriction, inflammation, and thrombosis.

Notably, Haffner et al. [3] showed that patients affected by diabetes without prior MI have a risk of CHD similar to non-diabetic patients with prior MI. As a result, the adult treatment panel of the National Cholesterol Education Program recognized T2DM as a CHD risk equivalent [4]. However, whether DM constitutes a risk equivalent to prior MI for cardiovascular (CV) mortality remains controversial. Prolonged duration of DM (> 10–12 years) increases CHD mortality in male diabetic patients at a rate similar to CHD mortality in male patients without diabetes but with a history of prior MI [5].

Therefore, there is a need to find specific markers for detecting different levels of disease severity or progression of T2DM associated with CHD. To this purpose, shifting focus from conventional risk factors and single disease biomarkers to biomarker ‘signatures’, composed of multiple disease-relevant proteins, would considerably enhance the management of complex diseases [6]. Indeed, employing a multimarker approach that assesses several biomarkers simultaneously, would enable the identification of multiple pathophysiological pathways, providing integrated information about the patient’s condition. Moreover, a multimarker strategy has the potential to enhance personalized patient care by identifying individuals at high risk of disease progression, thus facilitating stratification and preventive measures.

Over the past decade, technological advancements have introduced novel tools for the discovery and clinical implementation of prognostic, predictive, and diagnostic biomarkers for both CHD and DM. In this context, mass spectrometry (MS) has offered new approaches for the simultaneous quantitation of multiple protein biomarkers. The selected reaction monitoring (SRM)/multiple reaction monitoring (MRM) approach used in tandem MS on a triple quadrupole mass spectrometer, enables the simultaneous evaluation of peptides, derived from a protein of interest, with a high level of specificity and sensitivity [7]. The aim of this research was to assess, based on the above indicated innovative proteomic approach coupled to multivariate statistical analysis, the potential predictive role of multiple circulating proteins as biomarkers for the progression of cardiovascular damage associated with T2DM.

## Methods

### Patient recruitment

The study included 34 patients with both T2DM and CHD (group DC), 31 patients with T2DM without CHD (group DN) and 30 patients without diabetes but with a diagnosis of CHD (group NC), attending U.O.C. of Diabetology and Dietetics of Ulss 6 Euganea, Padova (Italy).

Patients affected by diabetes followed an isocaloric Mediterranean-style diet and received individualized hypoglycemic therapy. CHD was defined according to standard criteria and consulting anamnestic data from electronic medical and hospital records. Additionally, 90% of CHD patients were taking antihypertensive drugs. Subjects’ characteristics are shown in **Table ****S1**.

### Sample preparation

Blood samples were collected in citrate tubes (0.129 mmol/L) and plasma was immediately separated by centrifugation at 1500×*g* for 15 min at 4 °C. The plasma was then divided into aliquots, and frozen at -80 °C until analysis. Plasma samples were digested using the ProteinWorks™ eXpress Digest kit (Waters Corporation, Milford, MA, USA) according to the manufacturer’s instructions and as previously described [8]. Briefly, 35 μL of plasma were mixed with 12 μL of 200 μg/mL Intact mAb Mass Check Standard (Waters Corporation), used as an internal LC-MS standard for MS optimization and performance testing, and then diluted to a total volume of 120 μL using the digestion buffer provided in the kit. The resulting sample was added to a dried denaturant, placed into the dry block heater at 80 °C for 10 min. It was subsequently reduced and alkylated, digested with 30 μL of trypsin solution and placed into the dry block heater at 45 °C for 2 h. The trypsin inactivation agent was added to the sample and the supernatant was recovered by centrifugation and stored at − 80 °C for further clean-up using solid phase extraction (SPE). Protein digest clean-up was performed according to the ProteinWorks™ μElution SPE Clean-up protocol (Waters Corporation, Milford, MA, USA) using the Oasis μElution MCX plate. Before MS analysis, the purified plasma samples were combined with a mixture of stable isotope-labeled internal standards (SIS) for quantitation of a total of 125 proteins (peptides are reported in Table S2), provided with the Peptiquant Plus Human Plasma Proteomics Kit (Human BAK-125, Cambridge Isotope Laboratories, Inc., Tewksbury, MA, USA).

### Liquid chromatography mass spectrometry (LC-MS) analysis

Two microliters of each sample, containing a SIS mix 1.6x the lowest point on the curve (LPOC) for each protein, were injected into a Xevo TQ-S micro triple quadrupole mass spectrometer coupled to a Waters ACQUITY ultra-performance liquid chromatography (UPLC) M-Class system through an ionKey source (Waters Corporation, Milford, MA, USA). The instrument was operated in positive ion mode with unit resolution. The capillary voltage was set at 3.80 kV with the source temperature at 120 °C. After an isocratic trapping for 1 min at a flow rate of 30 μL/min with 99.5% solution A (99.9% LC-MS-grade water with 0.1% formic acid) and 0.5% solution B (99.9% LC-MS-grade acetonitrile with 0.1% formic acid) using an ACQUITY UPLC M-Class Symmetry C18 Trap Column, 100 Å, 5 μm, 300 μm × 50 mm (Waters Corporation, Milford, MA, USA), an iKey Peptide HSS T3 column, 100 Å 1.8 μm, 150 μm × 100 mm (Waters Corporation, Milford, MA, USA) was employed for peptide separation. The flow rate was set to 3 μL/min, and the column temperature was maintained at 60 °C. A gradient of solvent A and solvent B was applied with a total run time of 25 min as follows: 0–2.5 min at 2% B; 2.5–17.5 min with linear increase from 2 to 90% B; 17.5–19.5 min at 90% B; 20–25 min at 2% B. The analysis was conducted in duplicate. Transition and collision energy optimization were performed using Skyline software (version 4.2). The peptides and transitions of the multimarker 125-protein panel are reported in Table S2. A scheduled method based on peptide retention time was implemented to maintain the dwell time between 0.007 and 0.066 s. A pool of plasma sample was analyzed for quality controls throughout the run, and a coefficient of variation below 20% was considered acceptable (Table S3). A standard curve 0.04x–16x was run to test the linearity of the response for all the peptides using an increasing concentration of light peptides with a fixed amount of SIS (1.6x) in a digested synthetic serum. The standard data were analyzed with Skyline software using weighted linear regression with a weighting factor of 1/x and the accuracy of each standard point was evaluated. Moreover, the quality was further assessed by manually inspecting each peak in the data set using Skyline software and considering the relative dot product obtained by comparing transition distribution in light and heavy peptides. Peptide quantity is expressed as the ratio of the integrated area of the endogenous peptide to the area of the corresponding SIS calculated using Skyline. Missing values were handled as the default method in multivariate analysis of chemometric data, which replaces missing and zero values with a small value (the half of the minimum positive values in the original data) assuming to be the detection limit.

### Gene ontology analysis

The Search Tool for the Retrieval of Interacting Genes/Proteins (STRING 10.5) database [9] was employed to identify enriched Gene Ontology (GO) terms in the biological process, molecular function, or cellular component categories, as previously described [10]. The enrichment function of STRING, which calculates an enrichment *p* value based on a hypergeometric test according to the method of Benjamini and Hochberg for multiple testing correction (*p* value cut-off < 0.05), was used.

### Statistical analysis

The statistical analysis was performed using JMP® Version Pro 17 software for Windows (SAS Institute Inc, Cary, NC, USA) and MetaboAnalyst 5.0 [11]. To assess the statistical differences among groups, a preliminary parametric one-way analysis of variance (ANOVA) followed by post-hoc analysis with Fisher’s least significant difference (LSD) method were used. Fishers’s LSD method was chosen in order to preserve the experiment-wise type I error rate at the nominal level of significance and avoid exclusion of potentially interesting proteins from the initial protein panel. Differences were considered statistically significant when *p* < 0.05. Subsequently, several non-parametric multivariate methods were used, both unsupervised and supervised, each one providing additional integrated information, in order to classify samples from patients and evaluate the relevance of the analysed proteins’ panel, as hereafter indicated. The first classification algorithm was principal component analysis (PCA) [12, 13], an unsupervised method useful to identify the directions that best explain the variance in a complex data set, providing a summary based on fewer variables (scores) that represent the weighted average of the original variables, whose profiles are the loadings. The loading plot indicates the correlations between different variables; when the angle between eigenvectors is close to zero, the variables are positively correlated, while angles of 90° indicate no correlation and angles near 180° indicate negative correlations. As further approach, partial least squares-discriminant analysis (PLS-DA) was used [14], which consists in a supervised method based on multivariate regression technique to extract, with linear combination of the original variables, the information able to predict a class membership; PLS-DA also yields the variable importance in projection (VIP), that is a weighted sum of squares of the loadings, considering the amount of explained variation in each dimension, that indicates the feature importance. The sparse partial least squares-discriminant analysis (sPLS-DA) was utilized to reduce the number of variables and obtain an interpretable model in a single procedure, with strong predictive performance [15]. Agglomerative hierarchical cluster analysis (AHC), a non-parametric statistical approach based on dissimilarities of objects, was used to obtain data grouping displayed as dendrograms or heatmaps [16]; the computation considered Euclidean distance measure, with Ward’s linkage method clustering algorithm, which minimizes the sum of squares between clusters. Additional information for data classification was obtained with random forest method, a supervised learning algorithm for high dimensional data analysis which uses an ensemble of classification trees, originated by random feature selection from a bootstrap sample at each branch [17].

Receiver operating characteristic (ROC) curves were calculated to predict the ability of the selected proteins to classify subjects’ groups. ROC analysis was based on a nominal multivariable logistic regression considering the selected panel of proteins as potential predictors. The area under the ROC curve (AUC) indicated the model goodness of fit; a value of 1 indicates a perfect fit while a value near 0.5 suggests that the model cannot discriminate among groups. Predicted responses were calculated at optimal cut points for sensitivity (the proportion of true positives) and specificity (the proportion of true negatives) estimation.

## Results

Clinical and metabolic parameters in the three groups of subjects are presented in Table S1. Full information regarding patients’ cohort has been previously published [2]. T2DM patients (Group DN) had significantly higher values of fasting plasma glucose (FPG) and glycosylated hemoglobin (HbA1c) compared to non-diabetic patients with CHD (Group NC). No significant differences were found regarding the LDL cholesterol between diabetic and non-diabetic patients with CHD (DC vs. NC groups).

**Fig. 1:**
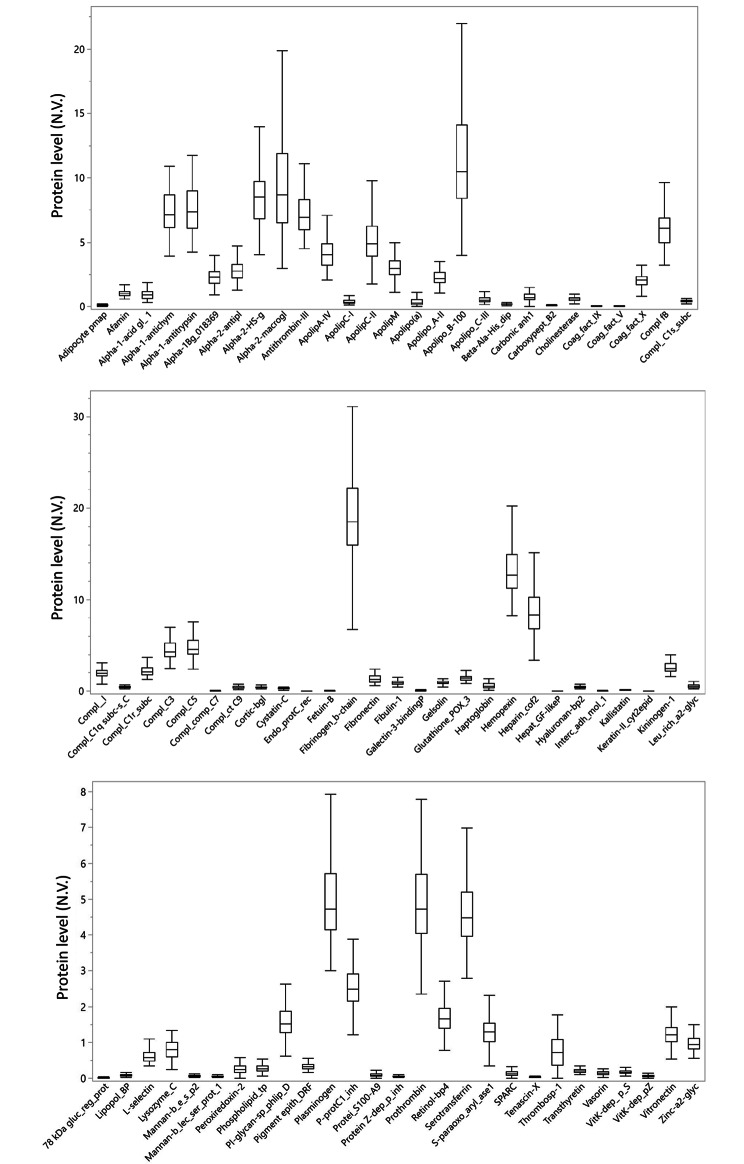
Box plot of the distribution of the 81 proteins analysed. The edges of the boxes indicate the 25th and 75th quantiles, including the middle 50% of the data; whiskers indicate the furthest points within 1.5 x IQR from the box. IQR is the interquartile range, defined as the difference between the 75th and 25th percentiles. For graphical purpose, the full names of the proteins have been abbreviated, as reported in Table S4. Protein level in y-axis is expressed as a normalized value (N.V.) on the basis of internal standard

Plasma samples from the subjects were analysed using a multiplexed targeted liquid chromatography mass spectrometry (LC-MS)-based assay, namely multiple reaction monitoring (MRM), to quantitatively determine a panel of proteins with potential roles in cardiovascular diseases (Table S2). The development of the MRM method was performed by adding stable isotope-labeled internal standards (SIS) to a pool of plasma samples, enabling the creation of a scheduled method to measure a total of 118 peptides corresponding to 118 proteins. Out of these, 81 proteins (Table S4; since the UniProt short name is not always available nor shorter than the full name, the table presents the abbreviated names/acronyms used in the present study, in order to ensure adequate display in the graphs) were successfully quantified in the human plasma samples. Peptides that were undetectable or lacked a linear response in the standard curve were excluded. The 81 considered proteins included members of lipoproteins, coagulation factors, complement system, as well as transport and signalling proteins. The distribution profile of these proteins in all subjects is presented in Fig. 1.

**Fig. 2:**
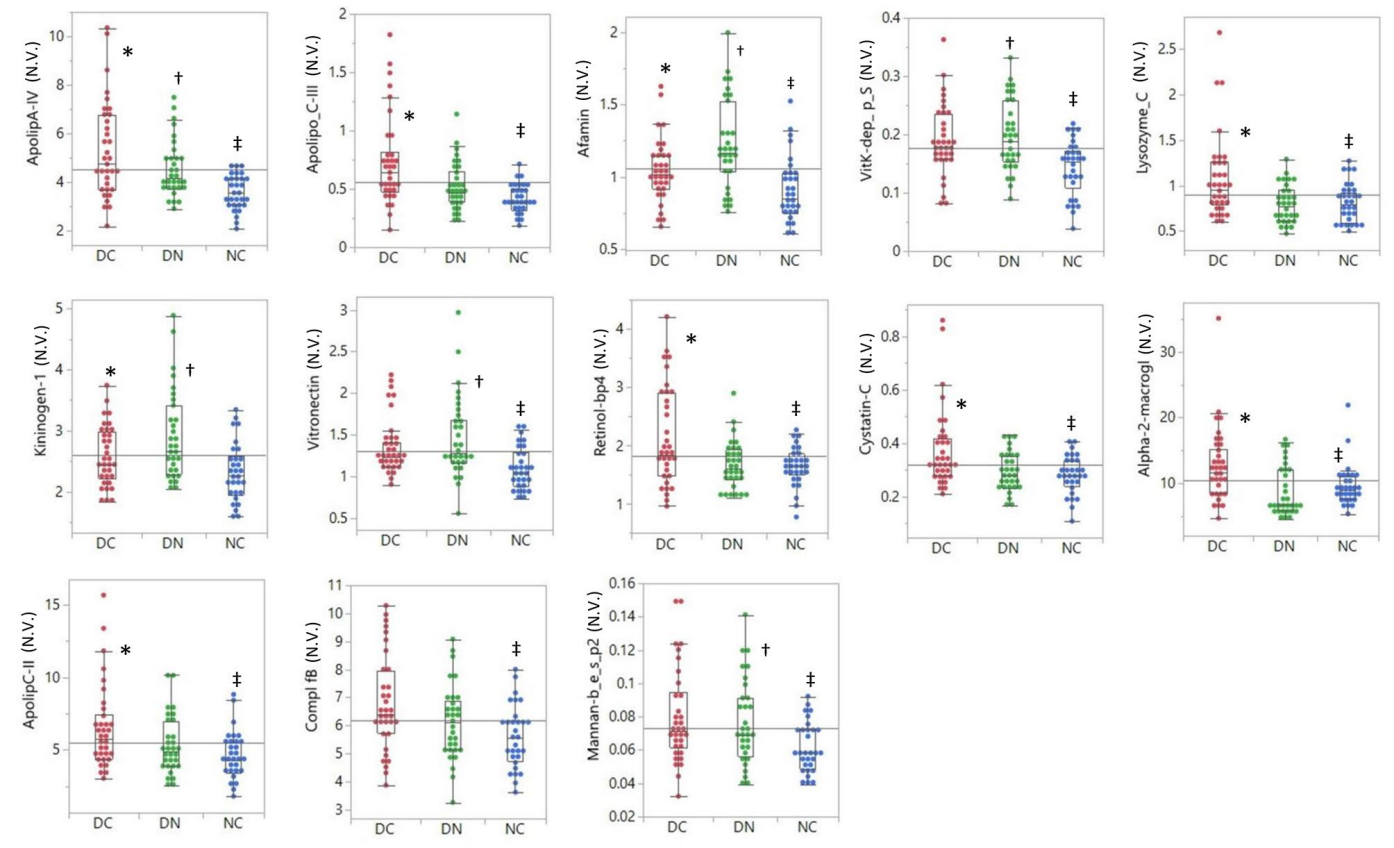
Box plot of the concentrations of the 13 significant proteins detected with ANOVA in the 3 groups of subjects (each group characteristics are indicated in Methods section). Refer to Table 1 for complete post-hoc comparisons between groups. The edges of the boxes indicate the 25th and 75th quantiles, including the middle 50% of the data; whiskers indicate the furthest points within 1.5 x IQR from the box. The continuous horizontal line in each graph represents the overall arithmetic mean of the data set. For graphical purpose, the full names of the proteins have been abbreviated, as reported in Table S4. Protein concentration in y-axis is expressed as a normalized value (N.V.) on the basis of internal standard. Asterisks indicate paired significant differences with Fisher’s Least Significant Difference (LSD) test at the 0.05 level or less. * comparison DC vs. DN; † comparison DN vs. NC; ‡ comparison DC vs. NC

A preliminary evaluation of the differences in the proteomic profile among patient groups was obtained with ANOVA. Thirteen of the 81 proteins analysed were found to be significantly different; Table 1 reports the statistical details, together with the between-group differences detected with Fisher’s LSD test. Figure 2 shows the distribution of significant data in the three groups of subjects.

**Table 1:** Significant features at ANOVA presenting the 13 proteins potentially of interest as discriminant among groups. ANOVA was followed by post-hoc analysis with Fisher’s least significant difference method (Fisher’s LSD) in order to detect the paired significant comparisons. See also Fig. 2 for graphical display. UniProt Accession Number is the protein stable identifier according to https://www.uniprot.org/

Protein	UniProt Accession Number	ANOVA F value	-log 10(p)	ANOVA p	Fisher’s LSD paired comparisons
Apolipoprotein A-IV	P06727	11.86	4.58	< 0.0001	DC-DN; DC-NC; DN-NC
Apolipoprotein C-III	P02656	11.70	4.53	< 0.0001	DC-DN; DC-NC
Afamin	P43652	11.25	4.37	< 0.0001	DN-DC; DC-NC; DN-NC
Vitamin K-dependent protein S	P07225	8.79	3.49	0.0003	DC-NC; DN-NC
Lysozyme C	P61626	8.06	3.23	0.0006	DC-DN; DC-NC
Kininogen-1	P01042	8.01	3.21	0.0006	DN-DC; DN-NC
Vitronectin	P04004	7.77	3.12	0.0008	DC-NC; DN-NC
Retinol-binding protein 4	P02753	7.50	3.02	0.001	DC-DN; DC-NC
Cystatin C	P01034	7.45	3.00	0.001	DC-DN; DC-NC
Alpha-2-macroglobulin	P01023	6.95	2.81	0.0016	DC-DN; DC-NC
Apolipoprotein C-II	P02655	5.44	2.23	0.0059	DC-DN; DC-NC
Complement factor B	P00751	5.30	2.18	0.0066	DC-NC
Mannan-binding lectin serine protease 2	P01034	5.22	2.15	0.0071	DC-NC; DN-NC

**Fig. 3:**
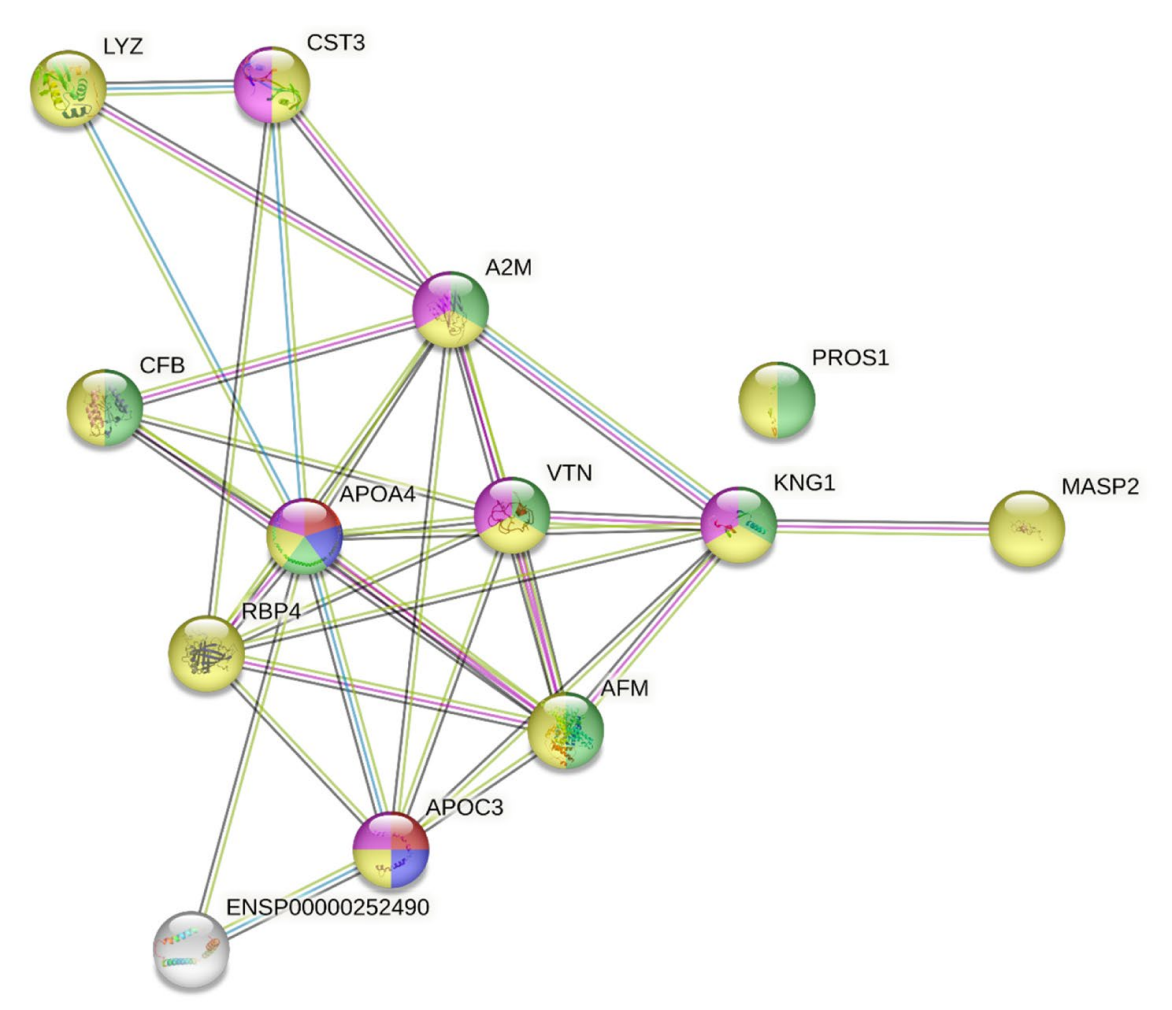
GO analysis of proteins. Enriched GO terms in the cellular component category are highlighted in different colors: yellow, extracellular exosome; violet, collagen-containing extracellular matrix; green, blood microparticle, and in the biological process: red and blue, chylomicron remodeling and assembly. See Table S5 and S6 for details

The GO analysis, specifically related to the cellular component term revealed the enrichment of several GO terms (Fig. 3 and Table S5). Notably, there was enrichment in terms associated with blood microparticles (*n* = 6, *p* = 1.38e-07, i.e., VTN, AFM, KNG1, A2M, APOA4, PROS1), extracellular exosomes (*n* = 11, *p* = 1.20e-06, i.e., VTN, AFM, APOC3, LYZ, KNG1, A2M, APOA4, RBP4, PROS1, CST3, MASP2), and the collagen-containing extracellular matrix (*n* = 6, *p* = 3.73e-05, i.e. VTN, APOC3, KNG1, A2M, APOA4, CST3). Furthermore, the GO analysis related to the biological process term revealed the enrichment of GO terms related to chylomicron remodeling and assembly (i.e. APOC3, APOA4) (Fig. 3 and Table S6).

**Fig. 4:**
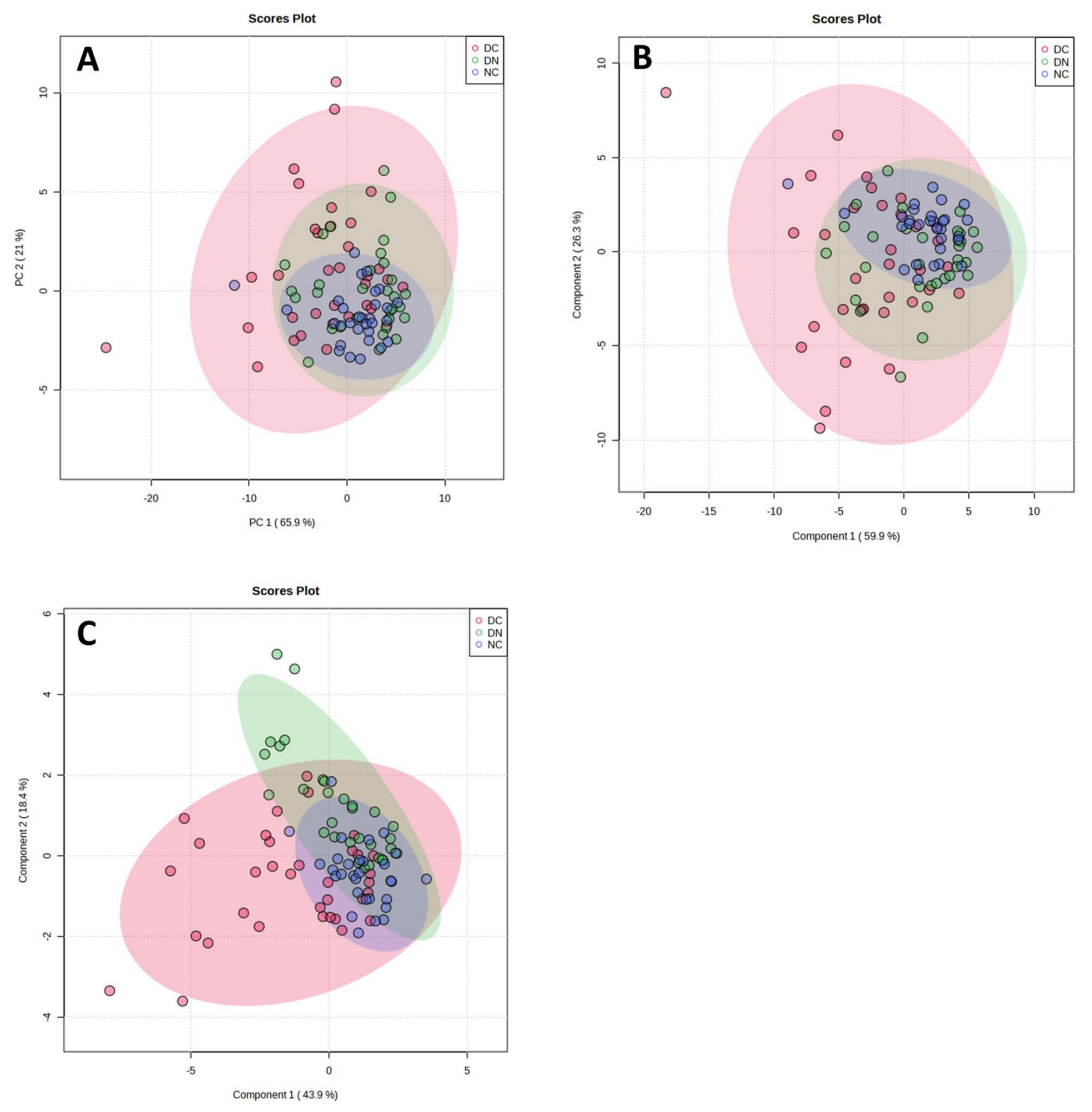
Scores plot between PCs, obtained on the 13 selected proteins with PCA (**panel A**), PLS-DA (**panel B**) and sPLS-DA (**panel C**). The explained variances for each component are shown in brackets

To assess the combined discriminatory ability of the selected proteins across all the three groups of patients, several multivariate approaches were undertaken. An unsupervised analysis using PCA did not show a net distinction of the groups (Fig. 4, **panel A**). The scores plot displayed overlapping clusters, with the DC group exhibiting the greatest dispersion. The supervised approaches provided by PLS-DA (Fig. 4, **panel B**) and sPLS-DA (Fig. 4, **panel C**), which can predict the class membership, provided results consistent with PCA. Although sPLS-DA suggested a slightly improved separation of the DC group, the distribution remained mixed and overlapped with the other two groups. In addition, both PLS-DA and sPLS-DA confirmed the proteins that were most effective at distinguishing among the groups (**Figure ****S1**** panels A, B**), a finding corroborated by the random forest supervised learning algorithm (**Figure ****S1**** panel C**). Notably, a good agreement emerged between the features identified using ANOVA (parametric) and those identified through non-parametric procedures (Table S7).

**Fig. 5:**
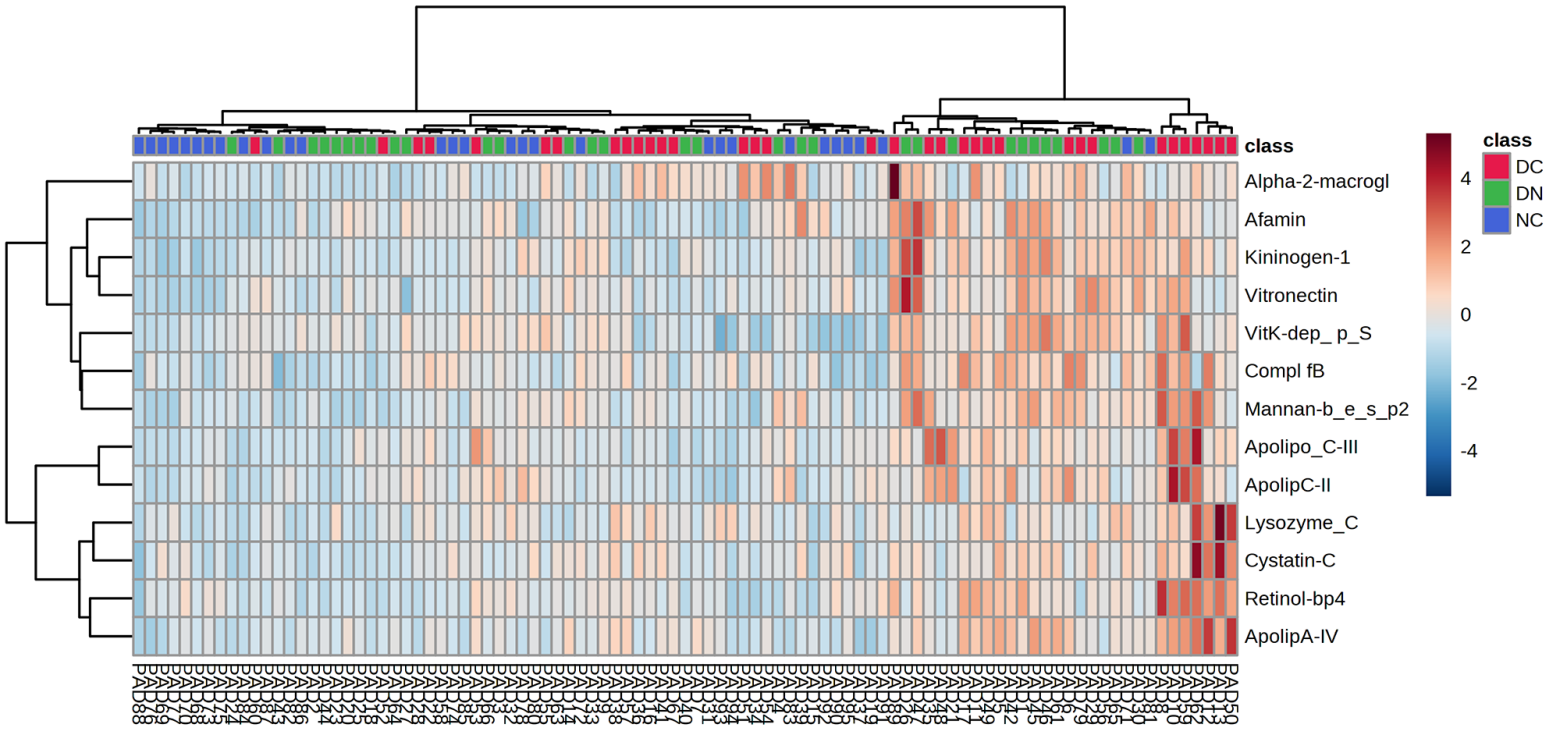
Clustering result shown as heatmap (distance measure using Euclidean, and clustering algorithm using Ward method), obtained on the 13 selected proteins significant at ANOVA. Heatmap color intensity is proportional to the parameter’s value (blue to red), as in the reported scale on the right of the figure

**Fig. 6:**
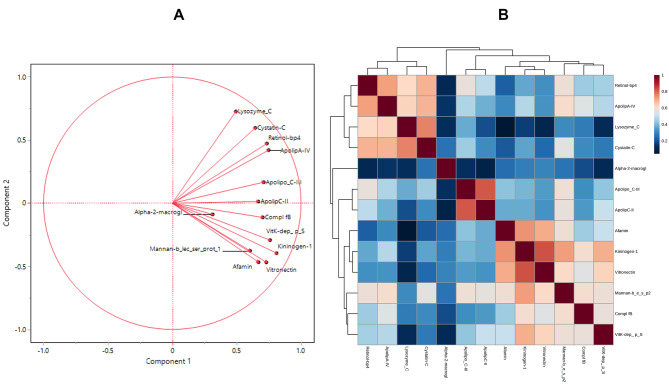
**A**) Loading plot from PCA on correlations obtained with the 13 selected proteins significant at ANOVA. **B**) Heatmap showing the mutual correlation between the proteins

Cluster analysis was also conducted using the 13 selected proteins, as depicted in the comprehensive heatmap shown in Fig. 5. Despite a not optimal separation of the three groups of subjects, confirming the results obtained from the other multivariate methods, an interesting pattern of proteins emerged. A first main cluster comprises apolipoprotein C-III, apolipoprotein C-II, apolipoprotein A-IV, retinol-binding protein 4, lysozyme C and cystatin-C; the second one includes, albeit with sub-grouping, the remaining proteins, i.e. alpha 2 macroglobulin, afamin, kininogen 1, vitronectin, vitamin K-dependent protein S, complement factor B and mannan-binding lectin serine protease 2 (Fig. 5). For a general view, the heatmap showing the pattern of the 13 selected proteins without group cluster ordination is shown in Figure S2, while the overall grouping of the entire panel of 81 proteins as determined by cluster analysis is presented in Figure S3. Figure S2B illustrates the clustering results also including additional factors, such as age, gender, lipid profile and glucose parameters, confirming the clustering of the selected panel of proteins. Additional information on the relative correlation among proteins derives from the angles between the vectors in the PCA loading plot of Fig. 6**panel A**, considering that when two vectors form a small angle, then the two corresponding variables are positively correlated, while when the vectors are placed at 90°, they are not likely correlated. For example, the cluster comprising apolipoprotein A-IV, retinol-binding protein 4, lysozyme C and cystatin C, previously identified through cluster analysis, also exhibits a close relationship here (Fig. 6**panel B**).

**Fig. 7:**
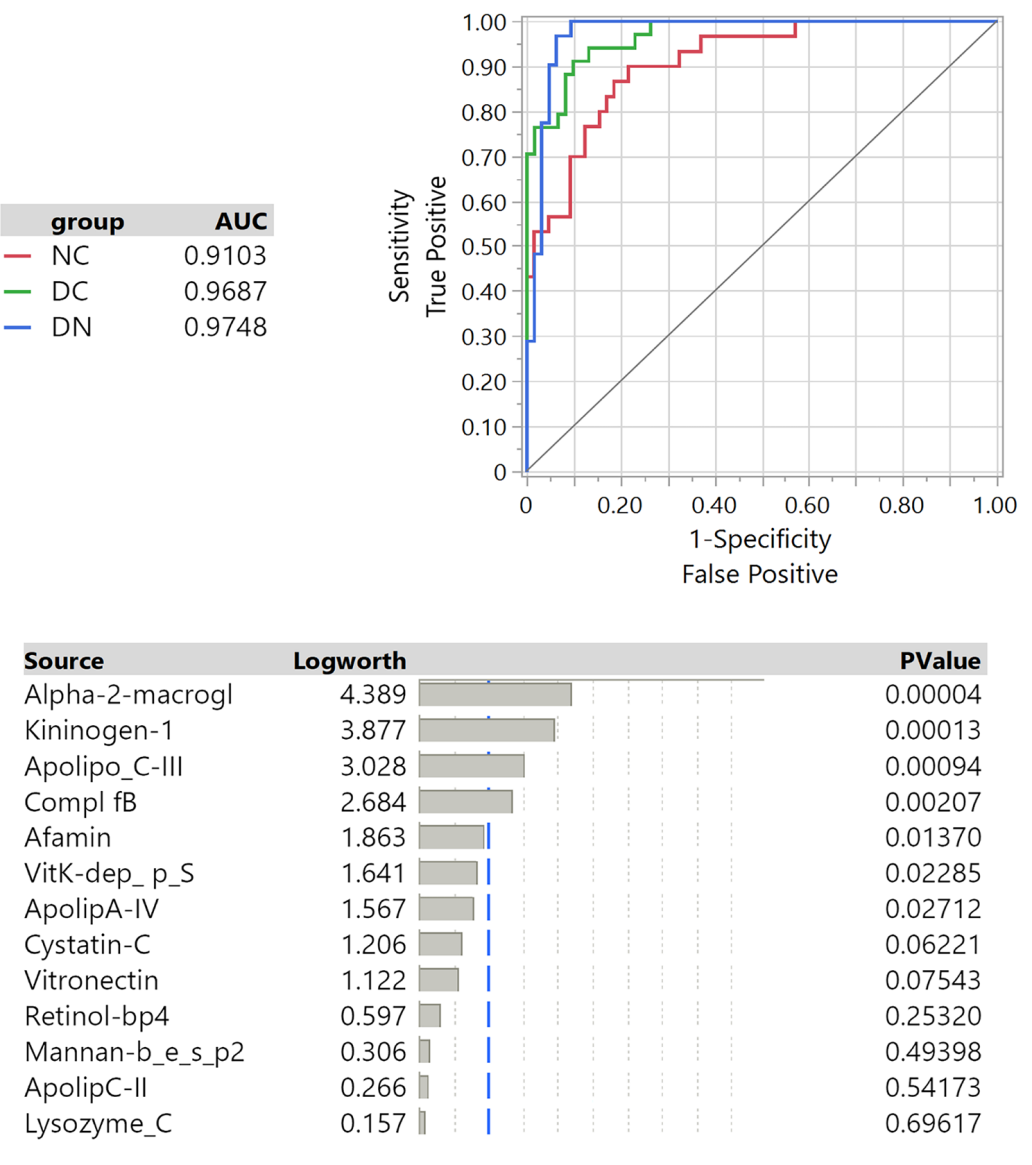
Upper panel: ROC analysis obtained with nominal logistic plot of the 13 selected proteins; the AUC for each respective curve is indicated. Lower panel: the table presents the effect summary with logworth and *p* value for each item. Whole model test was successfully accomplished with overall significance of *p* < 0.0001

**Fig. 8:**
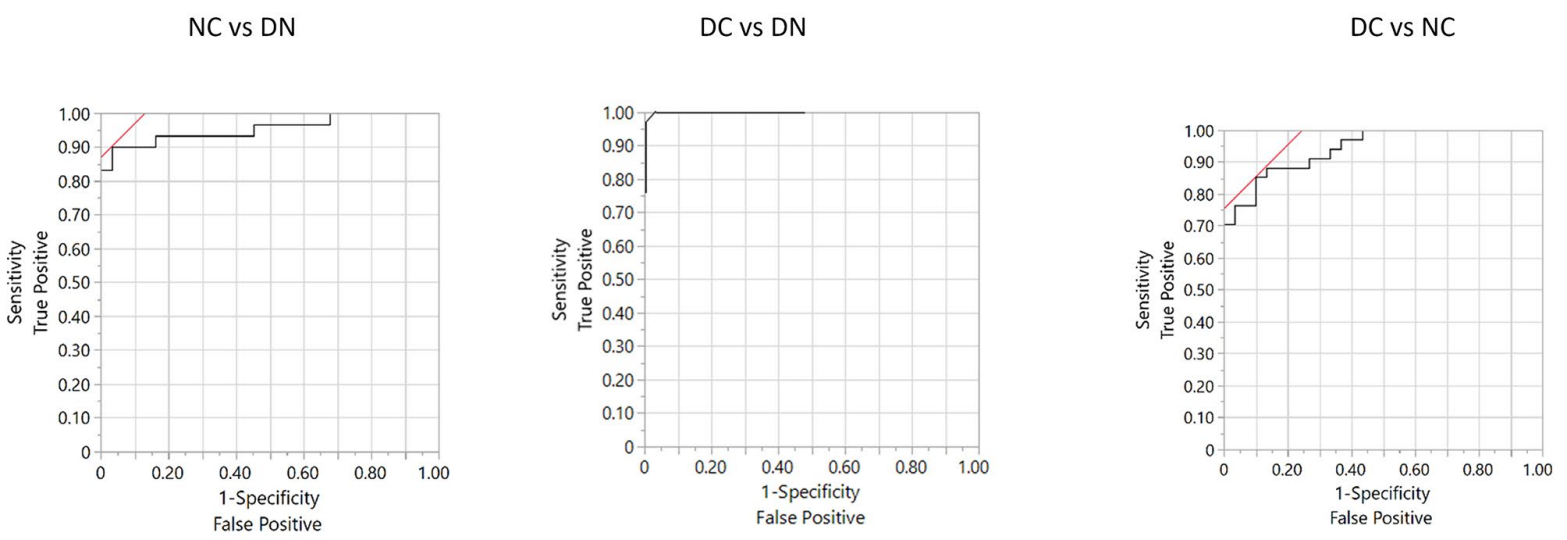
ROC analysis obtained with nominal logistic plot of the 13 selected proteins, according to paired comparisons, considering DN group or NC group as reference; the AUC for each respective curve is indicated in Table 2

To evaluate the diagnostic performance of the 13 selected proteins in distinguishing among the three groups of subjects, ROC curves were obtained following a nominal logistic regression. Figure 7**(upper panel)** indicates a significant overall distinction (whole model test *p* < 0.001) among the three groups of subjects, with AUC values ranging from 0.91 to 0.97, confirming the excellent goodness of fit for the model. The features that most contributed to the model are presented in Fig. 7**(lower panel)**. ROC analysis was further extended to evaluate paired group comparison, as shown in Fig. 8. Notably, the performance of the model, which incorporates the 13 selected proteins to discriminate between different pathological conditions, was remarkably strong, as documented by the excellent estimated discriminating parameters of sensitivity and specificity (Table 2). Table S8 provides a comprehensive summary of the effects observed in the paired comparisons. A further evaluation of the logistic nominal fitting procedure, excluding the features that did not result as significant in the regression with the 13-protein panel, did not improve the performance of the model, but conversely led to inferior specificity and sensitivity results (data not shown). This underscores the value of the comprehensive 13-protein model in optimizing the discriminating process.

**Table 2:** Performance of the model including the 13 selected proteins in discriminating the different pathological conditions in terms of sensitivity and specificity

	NC vs. DN	DC vs. DN	DC vs. NC
AUC	0.95	1.00	0.94
Sensitivity, %	90	100	85
Specificity, %	97	100	90
PPV, %	96	100	91
NPV, %	91	100	84
FDR, %	4	0	9
Probability threshold	0.577	0.994	0.561

AUC: Area under the curve; Sensitivity: [True positives/(True positives + False negatives)]; Specificity: [True negatives/(True negatives + False Positives)]; PPV: positive predictive value or precision, calculated as [True positives/(True positives + False positives)], or (1-FDR); NPV: negative predictive value, calculated as [True negatives/(True negatives + False negatives)]; FDR: False discovery rate, equivalent to [False positives/(False positives + True positives)]

## Discussion

The present study allowed us to measure, by means of a targeted mass spectrometry approach based on multiple reaction monitoring (MRM), multiple circulating proteins as possible biomarkers of cardiovascular damage progression associated with T2DM. Multivariate statistical evaluation of a panel of 81 simultaneously quantified plasma proteins permitted to identify 13 proteins of interest that were able to differentiate among subjects with diabetes mellitus, with or without CHD, and subjects with CHD but without diabetes. Gene Ontology analysis permitted to assess, as potential source of the identified circulating markers, both cellular component terms related to blood microparticles, extracellular exosomes and collagen-containing extracellular matrix, while under the biological process aspect, an enrichment of chylomicron remodelling and assembly term was detected, all suggesting complex interesting links among the identified protein pattern expression. The various statistical approaches conducted on the analytical data permitted to demonstrate the discriminating ability of the identified proteins, which were able to provide excellent classification results when taken as a whole panel, providing excellent ROC curve performances with a high degree of sensitivity and specificity.

Regarding the interrelationships suggested by the identified panel of proteins, it is possible to focus on their evidenced clustering behaviour, which helps to outline some specific patho-physiological aspects of the cardiovascular damage occurring with the progression of diabetic disease.

Lysozyme C, cystatin-C and retinol binding protein, which are the proteins grouped in one of the major clusters derived from cluster analysis (Fig. 5), and confirmed by loading plot results in PCA analysis (Fig. 6), have previously been proposed as markers of autoimmune-rheumatologic disorders [18]. These proteins probably may reflect a general reactivity of the organism, which in the subject affected by diabetes may drive to multiorgan damage. Recent observations suggest that T2DM is characterized by the presence of chronic inflammatory reaction in response to elevated blood glucose levels, also boosted by mediators of inflammation released by adipocytes and macrophages from adipose tissue [19]. It is not surprising that a sustained inflammatory state is a common pathogenetic factor for arterial diseases [20]; despite the traditionally well-documented alteration in lipoprotein metabolism, which causes retention of lipids in the intimal space of vessels, a low-grade chronic inflammation leads to attraction of cells of the immune system into the atherosclerotic plaque, accelerating atherosclerosis. The observation that in the present study the mean values of the above-mentioned proteins in DC subjects is higher than in DN patients (Fig. 2) supports the pathogenetic role in cardiovascular pathology development. Conversely, NC subjects already affected by cardiovascular disease, have a reduced level of the mentioned proteins; this reduction could be either related to a natural stabilization of the disease or to the effects of pharmacological therapy.

In particular, cystatin C, a member of the cystatin superfamily of cysteine protease inhibitors, has been linked to immune responses to exogenous and endogenous antigens since its encoding gene is modulated by various cytokines during infection and inflammation conditions [21]. In addition, cystatin C has various immunomodulatory functions through the control of the activity of cysteine proteases. Serum cystatin C has been shown to be a risk factor for CAD after the observation that cystatin C independently predicted major cardiovascular events, chronic kidney disease, and cardiovascular and all-cause mortality [22]. Animal studies have shown that cystatin C could interact with the inflammatory process, leading to activation of cathepsins which induce degradation of collagen in the atheromatous plaque, with an increased risk of rupture [23]. Moreover, cystatin C has been associated with the severity of CAD and has been suggested to independently predict the presence of vascular disease in subjects affected by DM with preserved renal function [24].

The role of apolipoprotein A-IV, which in the analysis also localizes close to the above-described protein cluster, deserves further attention. Apolipoprotein A-IV is considered a multifunctional protein with a protective role against atherosclerotic damage, as well as a modulator of glucose and lipid homeostasis [25]. However, there are observations that correlate high levels of apolipoprotein A-IV with kidney dysfunction [26, 27], although with no still definite role in this disease [25]. Moreover, apolipoprotein A-IV has been proposed as an early diagnostic biomarker in diabetes conditions [28], since the protein has been found inversely associated with prediabetes, suggesting a protective role in the disease. Therefore, the meaning of this protein is enhanced by the fact that a glycated form of apolipoprotein A-IV has been demonstrated to induce atherogenesis in patients affected by T2DM and with CHD [29]. The inclusion of the protein in a panel of potential markers of diabetes complications finds therefore a rational support.

Apolipoprotein C-II and apolipoprotein C-III, found close to each other in our analysis (Figs. 5 and 6), highlight the connection between lipid dysfunction and diabetes-related complications. These apolipoproteins, known for maintaining lipid balance and regulate triglyceride hydrolysis through lipoprotein lipase, have been recently implicated in controlling the transfer between VLDL and HDL subfractions, possibly affecting cardiovascular risk [30]. A possible link with the behaviour of the complement factor B, located close to the above two proteins in the PCA loading plot, is provided by the observations that despite complement factor C3 is linked to increased hyperlipidemia, conditioning a more atherogenic profile, on the contrary, complement factor B is not [31]; therefore, a less atherogenic profile may be activated, influencing the progress of cardiovascular damage. A few decades ago, it has been found [32] that chromosome 19 contains both the gene *APOC2* for apolipoprotein C-II and the gene *C3* for complement factor C3, delineating a possible common feature between the complement system and lipoprotein turnover. As a matter of fact, an increased level of complement factor C3 is correlated with serum cholesterol and triglycerides levels [33], and the C3 system is activated by Factor B [34]. Moreover, among the pathogenetic roles of the substance, a study by Varghese et al. [35] demonstrated that complement factor B, together with retinol binding protein, could be urinary markers for the prediction of glomerular disease, as found in diabetic nephropathy. Regarding apolipoprotein C-III, the possible pathogenetic role in diabetes-related complications is highlighted by the finding that the total apolipoprotein C-III level is enhanced in a condition of insulin resistance as in T2DM [36, 37]. Apolipoprotein A-III is upregulated by glucose, and therefore it has been suggested as a new target for the treatment of insulin resistance, besides the control of dyslipidemia [38].

Among the panel of the 13 proteins detected, the second major cluster identified in the present study by cluster analysis and confirmed by PCA, comprises afamin, kininogen-1, mannan-binding lectin serine protease 2, vitamin K-dependent protein S and vitronectin (Figs. 5 and 6). Afamin, first described as the fourth member of the human albumin gene family, is a glycoprotein similar to human albumin (55% amino acid sequence similarity) [39] mainly synthesised by the liver. The (patho-)physiological functions of afamin are still largely unknown, but the results of a study performed in mice overexpressing the human afamin gene indicate a possible causal role of the protein in the development of T2DM [40]. Further, epidemiological studies on more than 5,000 subjects demonstrated that plasma afamin is a predictor for the prevalence and the incidence of metabolic syndrome [40]. This finding was substantiated in a pooled meta-analysis of more than 20,000 individuals from eight prospective cohort studies that demonstrated an association of afamin plasma levels with the prevalence and incidence of T2DM [41]. Afamin is also a specific binding protein for vitamin E which has been associated with various diseases, among them obesity and T2DM [42]. Lastly, the urinary afamin to creatinine ratio has been proposed as a useful marker to predict patients with T2DM at high risk of nephropathy before the development of macroalbuminuria or reduced kidney function [43]. However, the causality of afamin’s association with diabetes mellitus and possible underlying mechanisms have still to be elucidated. Our data confirm the possible role of afamin in evaluating diabetes complications, being elevated in DN subjects, but reduced in both DC and NC subjects, indicating that elevated levels could predict the future progression of diabetes-related cardiovascular disease.

We also found that mannan-binding lectin serine protease 2 (MASP-2) is increased in the DN and DC groups. It is involved in the complement system activation. Upon recognition of a specific ligand the mannose-binding lectin (MBL)-associated serine protease 1 (MASP-1) undergoes structural rearrangement, which initiates the pathway by activating a second MBL-associated serine protease (MASP-2). The active MASP-2 is able to cleave the complement proteins C4 and C4-bound C2, thereby generating a C3-convertase and initiating the complement cascade [44]. Beyond its beneficial antimicrobial role, however, the MBL system has been associated with increased autoreactivity during several diseases [44], including diabetes.

The cleavage of kininogen, a precursor of bradykinin, occurring by mannose binding lectin-associated serine proteases (MASP 1 and 2) activity, contributes to the pro-inflammatory effect [45], and MASP-2 can initiate the complement cascade. Again, a link of the inflammatory system with a pathogenetic role in cardiovascular disease emerges also with these proteins here detected in the investigated groups, and confirmed by the findings by Hansen et al. [46] who suggested a role of mannose-binding lectins (MBL, which form multimolecular complexes with serine proteases [47]) as prognostic information on the diabetes-induced risk of mortality.

Vitamin K-dependent proteins (VKDPs), besides their role as clotting factors involved in the coagulation cascade, can also participate in the process of vascular calcification. Deposition of calcium phosphate in vessels is a common feature of diabetes complications [48], and the process of vascular smooth muscle transformation into osteocyte-like cells can be influenced through activation by several factors, among them inflammation, hypertension, and oxidized LDL [49, 50]. Matrix Gla protein (MGP) is an extracellular VKDP which inhibits vascular calcification, and a deficiency in vitamin K might deregulate its expression, conditioning vascular calcification [50]. MGP, through binding to vitronectin, the latter being a protein able to inhibit apoptosis, can affect cellular differentiation [51], therefore influencing vascular integrity. Protein S specifically, has been recognized to have anticoagulant activity, as well as a regulatory role in immune and vascular systems [52], supporting the possible role in modulating vascular health, and suggesting attention for the status of vitamin K in the subject, as a mechanism to ensure vascular functionality and integrity.

The strength of the present targeted mass spectrometry approach is the identification of circulating proteins as possible biomarkers of cardiovascular damage progression associated with T2DM, with excellent classification results in terms of sensitivity and specificity. The present study has some limitations. The first is the relatively small number of participants; this derives from the fact that plasma of the patients came from a previous sampling protocol related to our previous investigation [2] which had the character of a preliminary study, with no a priori power analysis. Another limitation comes from the fact that the included subjects are patients attending the local outpatient diabetes clinic, and therefore the sample is representative of a restricted geographical area. Moreover, we could not evaluate every possible lifestyle factor or comorbidity, and the observational structure of the study leaves the possibility of residual confounding factors. Another limitation regards the selection of the significant proteins at the univariate analysis, conditioning the fact that the model may not be robust, and more prone for overfitting. The selection was dictated by the preliminary evaluation of the entire 81-protein panel which did not provide a substantial distinction of the three groups of patients, as indicated for instance by cluster analysis of Figure S3. Also, a complete evaluation on 81-protein panels by PCA, PLS-DA and sPLS-DA (data not shown) again, did not provide useful patterns according to the three groups of patients. This suggested that possibly most of the initial panel proteins were unable to differentiate among patients, and for this reason, the analysis was focused on the ANOVA significant data only. However, the following parametric analysis with multivariable logistic regression and ROC curve evaluation suggested a promising data mining strategy for identifying possible disease markers, since the high specificity and sensitivity obtained. The significance obtained with the present data appears clear, although it deserves a confirmation with a larger sample size; an external validation was not possible at this stage, due to the limited number of observations, with the risk of over-extrapolating the performance of the model. All these aspects will be evaluated in a future more extensive experimental protocol.

Lastly, we recognise that our approach is not fully comprehensive as specific protein candidates have been measured. Considerable efforts are being leveraged year over year to discover and validate protein biomarkers using MS-based quantitative approaches for enhanced disease monitoring, companion diagnostics, and improved patient outcomes. However, although hundreds of MRM-based assays have been published, the information is dispersed throughout the literature, and protocols for the characterization of assay performances have not been standardized, making it difficult to evaluate the quality of published assays and, by extension, the results of those assays. As a result, despite the widespread capability to perform MRM assays, the benefits of MRM have not yet been fully realized by the biological and clinical research communities. For all these reasons, in this exploratory research, we used the optimized PeptiQuant Plus, with the biomarker assessment line designed for the precise and rapid quantitation of candidate disease markers (e.g., cardiovascular, cancer, etc.) that are suitable for biomarker discovery and verification studies, and that has been rigorously characterized according to the complete set of CPTAC (Clinical Proteomic Tumor Analysis Consortium) guidelines.

As a concluding remark, the present investigation, thanks to the availability of a cutting-edge proteomic tool, followed by multivariate data analysis, permitted the identification of a detailed panel of plasma proteins, which can give useful links in order to outline the pathophysiological history of cardiovascular complications in diabetes mellitus. The fact that DN patients, affected by diabetes but without CHD, have a short history of diabetes, here represented the optimal comparison term for catching the proteomic pattern before the onset of cardiovascular complications, which occur within several years, as documented by the DC group. Further investigations will provide validation on the prognostic role of the single proteins suggested by the present study. At present, the identified proteins taken as a reference panel, thanks to their evidenced high specificity and sensitivity, may represent a starting point to offer a new biomarker tool for the diagnosis and follow-up of diabetes-related complications.

### Electronic supplementary material

Below is the link to the electronic supplementary material.

**Supplementary Material 1: Table S1**. Clinical and metabolic parameters of subjects in study. **Table S2**. Peptides and transitions of the multimarker panel. **Table S3**. The pool of plasma sample analyzed for quality controls throughout the run. **Table S4**. List of the 81 proteins of the final biomarker panel, with UniProt Accession Number (https://www.uniprot.org/help/accession_numbers) and abbreviated names used for graphical display. **Table S5**. Gene Ontology (GO) analysis related to cellular component. **Table S6**. GO analysis related to the biological process revealing the enrichment of GO terms related to chylomicron remodeling and assembly. **Figure S1**. Validation of the 13 proteins selected with ANOVA. Panel A: Plot showing Important features identified by PLS-DA. The colored boxes on the right indicate the relative concentrations of the corresponding metabolite in each group under study. Variable Importance in Projection (VIP) is a weighted sum of squares of the PLS loadings taking into account the amount of explained variation in each dimension. Panel B: Plot showing the variables selected by the sPLS-DA model for a given component. The variables are ranked by the absolute values of their loadings. Panel C: Significant features identified by Random Forest. The features are ranked by the mean decrease in classification accuracy when they are permuted. **Table S7**. Summary of the features of the 13 proteins identified by ANOVA, according to the different non-parametric employed statistical procedures. The relevance of the proteins is indicated with semi-quantitative approximate scoring. **Figure S2**. A) Clustering result shown as heatmap (distance measure using Euclidean, and clustering algorithm using Ward method), obtained on the 13 selected proteins significant at ANOVA; differently from Figure 5, no ordering of groups was here applied, to show the overall pattern of protein distribution among groups. Heatmap color intensity is proportional to the parameter’s value (blue to red), as in the reported scale on the right of the figure. B) Clustering result including also factors such as age, gender, lipid profile, and glucose parameters. **Figure S3**. Clustering result shown as heatmap (distance measure using Euclidean, and clustering algorithm using Ward method), obtained on the overall panel of 81 proteins. **Table S8**. Effect summary of nominal logistic plot for three paired comparisons between groups of subjects by using the 13 selected proteins. Logworth and *p* value for each item are presented. Whole model test was successfully accomplished in any cases with overall significance of *p*<0.0001.

## Data Availability

The datasets used and/or analysed during the current study are available from the corresponding authors on reasonable request.
