# Supplementary material for: Multiplexed MRM-based proteomics for identification of circulating proteins as biomarkers of cardiovascular damage progression associated with diabetes mellitus
Source: Cardiovasc Diabetol. 2024 Jan 20;23:36. doi: 10.1186/s12933-024-02125-1 (PMC10800045; doi:10.1186/s12933-024-02125-1)
Supplement: Supplementary file 1 — Supplementary Material 1: Table S1. Clinical and metabolic parameters of subjects in study. Table S2. Peptides and transitions of the multimarker panel. Table S3. The pool of plasma sample analyzed for quality controls throughout the run. Table S4. List of the 81 proteins of the final biomarker panel, with UniProt Accession Number (https://www.uniprot.org/help/accession_numbers) and abbreviated names used for graphical display. Table S5. Gene Ontology (GO) analysis related to cellular component. Table S6. GO analysis related to the biological process revealing the enrichment of GO terms related to chylomicron remodeling and assembly. Figure S1. Validation of the 13 proteins selected with ANOVA. Panel A: Plot showing Important features identified by PLS-DA. The colored boxes on the right indicate the relative concentrations of the corresponding metabolite in each group under study. Variable Importance in Projection (VIP) is a weighted sum of squares of the PLS loadings taking into account the amount of explained variation in each dimension. Panel B: Plot showing the variables selected by the sPLS-DA model for a given component. The variables are ranked by the absolute values of their loadings. Panel C: Significant features identified by Random Forest. The features are ranked by the mean decrease in classification accuracy when they are permuted. Table S7. Summary of the features of the 13 proteins identified by ANOVA, according to the different non-parametric employed statistical procedures. The relevance of the proteins is indicated with semi-quantitative approximate scoring. Figure S2. A) Clustering result shown as heatmap (distance measure using Euclidean, and clustering algorithm using Ward method), obtained on the 13 selected proteins significant at ANOVA; differently from Figure 5, no ordering of groups was here applied, to show the overall pattern of protein distribution among groups. Heatmap color intensity is proportional to the parameter’s value (blue to re [file 12933_2024_2125_MOESM1_ESM.pdf]

Table S1. Clinical and metabolic parameters of subjects in study.

	**DC (n=34)**	**DN (n=31)**	**NC (n=30)**
Gender (M/F)	31/3	22/9	22/8
Age (y)	65.4±8.3**	60.0±5.9††	65.6±6.0
Diabetes duration (y)	9.9±7.6***	1.9±0.9	---
BMI (kg/m^2)	29.8±3.8	29.2±3.4	26.8±5.1‡
FPG (mg/dl)	141.6±41.1	144.8±32.1†††	94.7±12.9‡‡‡
HbA1c (%)	7.0±0.8	6.9±0.8†††	5.8±0.2‡‡‡
Total cholesterol (mg/dl)	152±34***	191±35†††	155±40
LDL cholesterol (mg/dl)	82±30**	112±39††	86±31
HDL cholesterol (mg/dl)	42±9	48±10	53±16‡‡
Triglycerides (mg/dl)	161±79	139±72†	91±40‡‡‡

Data are expressed as mean ±SD.

For each continuous variable, symbols indicate significant differences between means by the Tukey's HSD test. ***p<0.001, **p<0.01, DC *vs* DN; $^{\dagger\dagger\dagger}p$<0.001, $^{\dagger\dagger}p$<0.01, $^{\dagger}p$<0.05, DN *vs* NC; ‡‡‡ p<0.001, ‡‡ p<0.01, ‡ p<0.05, DC *vs* NC.

Abbreviations: BMI, body mass index; FPG, fasting plasma glucose; HbA1c, glycated haemoglobin; HDL, high density lipoprotein; LDL, low density lipoprotein.

Full information regarding patients' cohort has been previously published in Piarulli et al. (2022). Methods used for metabolic parameters determination are described in the mentioned paper. [Piarulli F, Banfi C, Brioschi M, Altomare A, Ragazzi E, Cosma C, Sartore G, Lapolla A. The Burden of Impaired Serum Albumin Antioxidant Properties and Glyco-Oxidation in Coronary Heart Disease Patients with and without Type 2 Diabetes Mellitus. Antioxidants (Basel). 2022 Jul 30;11(8):1501. doi: 10.3390/antiox11081501]

Table S2. Peptides and transitions of the multimarker panel.

Protein Name	Uniprot Accession Number	Sequence	Fragment ion	Fragment Ion Charge	Light		SIS		Collision energy light	Collision energy SIS
					Precursor m/z	Product m/z	Precursor m/z	Product m/z		
78 kDa glucose-regulated protein	P11021	ITPSYVAFTPEGER	y12	2	783.8936	676.8277	788.8977	681.8318	22	22
Adipocyte plasma membrane-associated protein	Q9HDC9	LLEYDTVTR	y7	1	555.2955	883.4156	560.2996	893.4239	16	16
Adiponectin	Q15848	IFYNQQNHYDGSTGK	y13	2	591.2727	756.3291	593.9441	760.3362	17	17
Afamin	P43652	DADPDTFFAK	y7	1	563.7562	825.4141	567.7633	833.4283	16	16
Alpha-1-acid glycoprotein 1	P02763	NWGLSVYADKPETTK	y13	2	570.2895	704.8696	572.9609	708.8767	13	13
Alpha-1-antichymotrypsin	P01011	EIGELYLPK	y2	1	531.2975	244.1656	535.3046	252.1798	22	22
Alpha-1-antitrypsin	P01009	LSITGTYDLK	y7	1	555.8057	797.4040	559.8128	805.4182	16	16
Alpha-1B-glycoprotein_VAR_018369	P04217	LETPDFQLFK	b2	1	619.3268	498.2635	623.3339	502.2706	16	16
Alpha-2-antiplasmin	P08697	LGNQEPGGQTALK	b5	1	656.8464	771.4359	660.8535	779.4501	23	23
Alpha-2-HS-glycoprotein	P02765	FSVVYAK	y5	1	407.2289	579.3501	411.2360	587.3643	12	12
Alpha-2-macroglobulin	P01023	AIGYLNTGYQR	y7	1	628.3251	851.4370	633.3293	861.4453	20	20
Antithrombin-III	P01008	DDLYVSDAFHK	y8	2	437.2068	483.7376	439.8782	487.7447	10	10
Apolipoprotein A-I	P02647	ATEHLSTLSEK	y10	2	405.8787	572.7959	408.5501	576.8030	11	11
Apolipoprotein A-II	P02652	EQLTPLIK	y4	1	471.2869	470.3337	475.2940	478.3479	13	13
Apolipoprotein A-IV	P06727	LGEVNTYAGDLQK	b3	1	704.3594	300.1554	708.3665	300.1554	28	28
Apolipoprotein B-100	P04114	FPEVDVLTK	y8	2	524.2897	450.7555	528.2968	454.7626	18	18
Apolipoprotein C-I	P02654	EWFSETFQK	y7	1	601.2798	886.4305	605.2869	894.4447	18	18
Apolipoprotein C-II	P02655	TYLPAVDEK	b2	1	518.2715	265.1183	522.2786	265.1183	15	15
Apolipoprotein C-III	P02656	GWVTDGFSSLK	b2	1	598.8009	244.1081	602.8080	244.1081	18	18
Apolipoprotein C-IV	P55056	ELLETVVNR	y4	1	536.8082	487.3075	541.8082	497.3075	16	16
Apolipoprotein D	P05090	VLNQELR	y5	1	436.2534	659.3471	441.2576	669.3554	13	13
Apolipoprotein E	P02649	LGPLVEQGR	y7	2	484.7798	399.7271	489.7840	404.7312	14	14
Apolipoprotein L1	O14791	VAQELEEK	y6	1	473.2480	775.3832	477.2551	783.3974	13	13
Apolipoprotein M	O95445	AFLLTPR	y5	1	409.2502	599.3875	414.2543	609.3958	12	12
Apolipoprotein(a)	P08519	TPAYYPNAGLIK	y7	1	654.3533	712.4352	658.3604	720.4494	23	23

Attractin	O75882	SVNNVVVR	y6	1	443.7589	700.4100	448.7630	710.4183	12	12
Beta-2-glycoprotein 1	P02749	ATVVYQGER	y6	1	511.7669	751.3733	516.7710	761.3816	14	14
Beta-Ala-His dipeptidase	Q96KN2	ALEQDLPVNIK	y5	1	620.3508	570.3610	624.3579	578.3752	22	22
Biotinidase	P43251	SHLIIAQVAK	b4	1	360.5572	451.2663	363.2286	451.2663	12	12
Cadherin-13	P55290	INENTGSVSVTR	b2	1	638.8282	228.1343	643.8324	228.1343	23	23
Carbonic anhydrase 1	P00915	VLDALQAIK	y7	1	485.8002	758.4407	489.8073	766.4549	14	14
Carboxypeptidase B2	Q96IY4	IAWHVIR	y4	2	298.8518	262.6729	302.1851	267.6729	12	12
Cathelicidin antimicrobial peptide	P49913	AIDGINQR	y6	1	443.7407	702.3529	448.7448	712.3612	15	15
Cation-independent mannose-6-phosphate receptor	P11717	GHQAFDVGQPR	y4	1	404.5354	457.2518	407.8715	467.2600	7	7
CD5 antigen-like	O43866	LVGGLHR	y5	1	376.2323	539.3049	381.2364	549.3131	13	13
Ceruloplasmin	P00450	IYHSHIDAPK	y8	2	394.2085	452.7354	396.8799	456.7425	11	11
Cholinesterase	P06276	YLTLNTESTR	y8	1	599.3091	921.4636	604.3133	931.4719	18	18
Clusterin	P10909	ELDESLQVAER	y3	1	644.8226	375.1987	649.8267	385.2069	20	20
Coagulation factor IX	P00740	SALVLQYLR	y5	1	531.8189	692.4090	536.8231	702.4173	16	16
Coagulation factor V	P12259	SEAYNTFSER	y8	1	602.2675	987.4530	607.2716	997.4613	18	18
Coagulation factor VIII	P00451	LHPTHYSIR	y7	2	375.2051	437.2325	378.5412	442.2366	12	12
Coagulation factor X	P00742	TGIVSGFGR	y5	1	447.2456	523.2623	452.2497	533.2706	15	15
Coagulation factor XII	P00748	EQPPSLTR	y6	2	464.2483	335.6978	469.2525	340.7019	13	13
Complement C1q subcomponent subunit B	P02746	IAFSATR	y5	1	383.2163	581.3042	388.2205	591.3125	13	13
Complement C1q subcomponent subunit C	P02747	FNA-VLTNPQGDYDTSTGK	y11	2	643.3059	584.7595	645.9773	588.7666	13	13
Complement C1r subcomponent	P00736	GLTLHLK	y5	2	261.1692	306.1974	263.8406	310.2045	2	2
Complement C1r subcomponent-like protein	Q9NZP8	VVVHPDYR	y7	2	328.8469	443.2325	332.1830	448.2366	8	8
Complement C1s subcomponent	P09871	TNFDNDIALVR	b2	1	639.3279	458.3085	644.3320	468.3168	23	23
Complement C2	P06681	HAFILQDTK	b4	1	358.1977	469.2558	360.8691	469.2558	9	9
Complement C3	P01024	TGLQEVEVK	y6	1	501.7769	731.3934	505.7840	739.4076	17	17
Complement C5	P01031	VFQFLEK	y5	1	455.7553	664.3665	459.7624	672.3807	12	12
Complement component C7	P10643	LIDQYGTHYLQSGSLG-GEYR	b2	1	753.0343	227.1754	756.3704	227.1754	26	26
Complement component C9	P02748	LSPIYNLVPVK	y9	2	621.8765	521.8184	625.8836	525.8255	16	16

Complement factor B	P00751	EELLPAQDIK	y6	1	578.3164	671.3723	582.3235	679.3865	16	16
Complement factor I	P05156	VFSLQWGEVK	y8	1	596.8217	946.4993	600.8288	954.5135	18	18
Corticosteroid-binding globulin	P08185	WSAGLTSSQVDLYIPK	y2	1	882.9620	244.1656	886.9691	252.1798	32	32
Cystatin-C	P01034	ALDFAVGEYNK	y6	1	613.8062	709.3515	617.8133	717.3657	19	19
Endothelial protein C receptor	Q9UNN8	TLAFPLTIR	y7	1	516.3160	817.4931	521.3202	827.5013	15	15
Fetuin-B	Q9UGM5	LVVLPFPK	y6	1	456.7995	700.4392	460.8066	708.4534	10	10
Fibrinogen alpha chain	P02671	ESSSHHPGIAEFPSR	y4	1	546.5937	506.2722	549.9298	516.2804	21	21
Fibrinogen beta chain	P02675	HQLYIDETVNS-NIPTNLR	b13	2	709.6992	764.3755	713.0352	764.3755	15	15
Fibrinogen gamma chain	P02679	YEASILTHDSSIR	y11	2	497.9195	600.3226	501.2555	605.3267	14	14
Fibronectin	P02751	HTSVQTTS-SGSGPFTDVR	y6	1	621.9659	734.3832	625.3020	744.3914	15	15
Fibulin-1	P23142	TGYYFDGISR	y6	1	589.7775	694.3519	594.7816	704.3601	18	18
Galectin-3-binding protein	Q08380	SDLAVPSELALLK	y8	1	678.3927	870.5295	682.3998	878.5437	18	18
Gelsolin	P06396	AGALNSNDAFVLK	b3	1	660.3513	1007.5156	664.3584	1015.5298	20	20
Glutathione peroxidase 3	P22352	QEPGENSEILPTLK	y12	2	777.9041	649.3535	781.9112	653.3606	22	22
Haptoglobin	P00738	DIAPTLTLYVGK	y9	2	645.8688	496.2948	649.8759	500.3019	17	17
Hemoglobin subunit alpha	P69905	VGAHAGEYGAEALER	y4	1	510.5829	488.2827	513.9190	498.2910	14	14
Hemopexin	P02790	NFPSPVDAAFR	y9	2	610.8066	480.2509	615.8107	485.2550	19	19
Heparin cofactor 2	P05546	TLEAQLTPR	y7	1	514.7904	814.4417	519.7945	824.4500	15	15
Hepatocyte growth factor-like pro-tein	P26927	SPLNDFQVLR	y7	1	594.8222	891.4683	599.8263	901.4766	21	21
Hyaluronan-binding protein 2	Q14520	VVLGDQDLK	y7	1	493.7795	788.4149	497.7866	796.4291	14	14
Ig mu chain C region	P01871	GFPSVLR	y5	2	388.2267	286.1817	393.2308	291.1859	10	10
Insulin-like growth factor I	P05019	GFYFNKPTGYGSSSR	y13	2	556.5986	732.3493	559.9347	737.3535	13	13
Inter-alpha-trypsin inhibitor heavy chain H2	P19823	SLAPTAAAK	y6	1	415.2425	558.3246	419.2496	566.3388	11	11
Intercellular adhesion molecule 1	P05362	LLGIETPLPK	y8	1	540.8368	854.4982	544.8439	862.5124	16	16
Interleukin-10	P22301	AHVNSLGENLK	y5	1	394.5473	560.3039	397.2187	568.3181	10	10
Kallistatin	P29622	VGSALFLSHNLK	y11	2	429.2470	593.8326	431.9183	597.8397	11	11
Keratin-type II cytoskeletal 2 epi-dermal	P35908	YEELQVTVGR	b2	1	597.3117	293.1132	602.3158	293.1132	21	21
Kininogen-1	P01042	TVGSDTFYSFK	y9	1	626.2982	1051.4731	630.3053	1059.4873	19	19

Leucine-rich alpha-2-glycoprotein	P02750	DLLLPQPDLR	y6	1	590.3402	725.3941	595.3444	735.4023	15	15
Lipopolysaccharide-binding protein	P18428	ITLPDFTGDLR	y8	1	624.3352	920.4472	629.3393	930.4555	20	20
L-selectin	P14151	AEIEYLEK	y6	1	497.7582	794.4294	501.7653	802.4436	14	14
Lysozyme C	P61626	AWVAWR	y4	1	394.7137	531.3038	399.7179	541.3121	11	11
Mannan-binding lectin serine protease 1	P48740	TGVITSPDFPNPYPK	b3	1	816.9170	258.1448	820.9241	258.1448	29	29
Mannan-binding lectin serine protease 2	O00187	WPEPVFGR	y5	1	494.2560	575.3300	499.2601	585.3383	17	17
Metalloproteinase inhibitor 2	P16035	EYLIAGK	y3	1	397.2264	275.1714	401.2335	283.1856	8	8
Myeloblastin	P24158	LVNVVLGAHNVR	y10	2	430.9262	539.8094	434.2623	544.8136	11	11
Peroxiredoxin-2	P32119	GLFIIDGK	y6	1	431.7553	692.3978	435.7624	700.4120	12	12
Phosphatidylinositol-glycan-specific phospholipase D	P80108	FGSSLITVR	y4	1	490.2822	488.3191	495.2863	498.3274	14	14
Phospholipid transfer protein	P55058	AVEPQLQEEER	y8	2	664.3281	514.7540	669.3322	519.7581	21	21
Pigment epithelium-derived factor	P36955	LQSLFDSPDFSK	y10	1	692.3432	1142.5364	696.3503	1150.5506	19	19
Plasma protease C1 inhibitor	P05155	FQPTLLTLPR	y8	1	593.3531	910.5720	598.3573	920.5803	15	15
Plasma serine protease inhibitor	P05154	AVVEVDESGTR	y9	2	581.2909	763.3581	586.2951	773.3663	20	20
Plasminogen	P00747	LFLEPTR	b2	1	438.2529	261.1598	443.2570	261.1598	12	12
Protein S100-A9	P06702	DLQNFLK	y5	1	439.2425	649.3668	443.2496	657.3810	12	12
Protein Z-dependent protease inhibitor	Q9UK55	ETSNFGFSLLR	y6	1	635.8250	692.4090	640.8291	702.4173	19	19
Prothrombin	P00734	ELLESYIDGR	y6	1	597.8037	710.3468	602.8078	720.3550	18	18
Retinol-binding protein 4	P02753	YWGVASFLQK	y8	1	599.8164	849.4829	603.8235	857.4971	17	17
Serotransferrin	P02787	DGAGDVAFVK	y7	1	489.7482	735.4036	493.7553	743.4178	15	15
Serum paraoxonase/arylesterase 1	P27169	IFFYDSENPPASEVLR	y8	1	942.4623	868.4887	947.4665	878.4970	34	34
Serum paraoxonase/lactonase 3	Q15166	IQNVLSEKPR	y8	2	395.2313	471.7720	398.5674	476.7761	16	16
SPARC	P09486	LEAGDHPVELLAR	y11	2	473.9245	589.3198	477.2606	594.3240	13	13
Tenascin	P24821	FTTDLDSPR	y7	1	526.2564	803.3894	531.2605	813.3976	15	15
Tenascin-X	P22105	ILISGLEPSTPYR	b2	1	723.4036	720.3675	728.4077	730.3758	26	26
Thrombospondin-1	P07996	GTLLALER	y4	1	436.7636	488.2827	441.7678	498.2910	15	15
Thrombospondin-4	P35443	KPQDFLEELK	y4	1	416.2274	518.2821	418.8988	526.2963	11	11
Thyroxine-binding globulin	P05543	AVLHIGEK	y6	2	289.5080	348.7056	292.1794	352.7127	7	7

Tissue factor pathway inhibitor (isoform 1)	P10646	FYYNSVIGK	y7	1	545.7820	780.4250	549.7891	788.4392	16	16
Transferrin receptor protein 1	P02786	GFVEPDHYVVVGAQR	y13	2	558.2862	734.8808	561.6223	739.8849	13	13
Transthyretin	P02766	GSPAINVAVHVFR	y11	2	456.2578	611.8564	459.5939	616.8605	9	9
Vascular cell adhesion protein 1	P19320	NTVISVNPSTK	y8	1	580.3195	845.4727	584.3266	853.4869	17	17
Vasorin	Q6EMK4	ESHVTLASPEETR	y5	1	485.9073	631.3046	489.2434	641.3128	16	16
Vitamin K-dependent protein S	P07225	SFQTGLFTAAR	y8	1	599.8144	836.4625	604.8185	846.4707	18	18
Vitamin K-dependent protein Z	P22891	GLLSGWAR	y5	1	430.2429	576.2889	435.2470	586.2971	12	12
Vitronectin	P04004	FEDGVLDPDYPR	y5	1	711.8304	647.3148	716.8346	657.3230	28	28
Zinc-alpha-2-glycoprotein	P25311	EIPAWVPFDPAAQITK	y10	1	891.9749	1087.5782	895.9820	1095.5924	26	26

Table S3. The pool of plasma sample analyzed for quality controls throughout the run.

Sample	Apolipoprotein C-III	Lysozyme C	Retinol-binding protein 4	Afamin	Alpha-2-macroglobulin	Apolipoprotein A-IV	Apolipoprotein C-II	Complement factor B	Cystatin-C	Kininogen-1	Mannan-binding lectin serine protease 2	Vitamin K-dependent protein S	Vitronectin
**Pool**	0.5590	0.8540		1.2341	11.6010	4.5707	5.1983	6.7125	0.2810	2.8171	0.0651	0.1593	1.3553
**Pool**	0.4649	0.6715	1.2959	1.0651	10.3459	3.4912	4.1933	5.5715	0.2168	2.3886	0.0442	0.1474	1.1611
**Pool**	0.4620	0.6462	1.2580	1.0201	9.1705	3.5974	4.0780	4.8233	0.2638	2.2260	0.0524	0.1310	1.0993
**Pool**	0.5189	0.7836	1.4175	1.1930	9.6188	4.2150	4.6121	5.7947	0.2931	2.3492	0.0595	0.1527	1.2542
**Pool**	0.4013	0.5589	1.3700	0.9188	8.0766	3.0048	3.5507	4.5222	0.2011	1.9410	0.0460	0.1137	0.9671
**Pool**	0.5600	0.9363	1.5666	1.3144	11.7619	4.2153	5.1975	6.4788	0.2513	2.6928	0.0689	0.1606	1.4158
**Pool**	0.4655	0.6750	1.2269	1.0466	9.0510	3.5315	3.9922	4.8449	0.2586	2.1929	0.0469	0.1406	1.1323
**Pool**	0.4900	0.6527	1.2253	1.0712	9.5217	3.7869	4.2435	5.5211	0.2769	2.1899	0.0535	0.1404	1.1942
**Pool**	0.6288	0.9116	1.6997	1.3639	12.1427	4.7918	5.3520	6.7060	0.2665	3.1027	0.0675	0.1674	1.4548
**Pool**	0.4517	0.6342	1.2486	0.9915	9.3066	3.4857	4.3369	5.0162	0.2451	2.1141	0.0510	0.1262	1.1297
**Pool**	0.4673	0.6854	1.2359	1.0593	9.0494	3.5842	4.3443	5.3143	0.2268	2.3277	0.0514	0.1420	1.1095
**Pool**	0.6847	1.0285	1.8629	1.4509	13.5675	5.4146	6.8727	7.7248		3.2669	0.0749	0.1987	1.7153
**Pool**	0.5252	0.7931	1.4571	1.1692	10.1401	4.2327	4.7694	6.0969	0.2667	2.5065	0.0594	0.1442	1.2709
**Pool**	0.4934	0.7438	1.3158	1.0584	9.6899	3.6942	4.1641	5.5315	0.2656	2.2638	0.0549	0.1510	1.1735
**Pool**	0.4409	0.7024	1.2053	0.9916	9.2452	3.2507	4.3614	5.1404	0.2232	2.1121	0.0496	0.1141	1.0830
**Pool**	0.5653	0.8011		1.2754	11.7300	4.7295	5.3707	6.2456	0.3172	2.7895	0.0662	0.1505	1.3593
**Pool**	0.3754	0.5364	1.0692	0.8727	7.6045	2.9478	3.3381	4.2555	0.1760	1.8193	0.0410	0.1088	0.9241
**%CV pool**	15.63427	18.07184	15.33524	14.32765	15.54673	17.35492	18.1385	16.01393	14.2500	16.41282	17.41677	15.32016	15.81651

Protein	% CV pool
**Apolipoprotein C-III**	15.63
**Lysozyme C**	18.07
**Retinol-binding protein 4**	15.34
**Afamin**	14.33
**Alpha-2-macroglobulin**	15.55
**Apolipoprotein A-IV**	17.35
**Apolipoprotein C-II**	18.14
**Complement factor B**	16.01
**Cystatin-C**	14.25
**Kininogen-1**	16.41
**Mannan-binding lectin serine protease 2**	17.42
**Vitamin K-dependent protein S**	15.32
**Vitronectin**	15.82

Table S4. List of the 81 proteins of the final biomarker panel, with UniProt Accession Number (https://www.uniprot.org/help/accession_numbers) and abbreviated names used for graphical display.

No.	Protein	Uniprot Accession Number	Abbreviated name
1	Adipocyte plasma membrane-associated protein	Q9HDC9	Adipocyte pmap
2	Afamin	P43652	Afamin
3	Alpha-1-acid glycoprotein 1	P02763	Alpha-1-acid gl_ 1
4	Alpha-1-antichymotrypsin	P01011	Alpha-1-antichym
5	Alpha-1-antitrypsin	P01009	Alpha-1-antitrypsin
6	Alpha-1B-glycoprotein_VAR_018369	P04217	Alpha-1Bg_018369
7	Alpha-2-antiplasmin	P08697	Alpha-2-antipl
8	Alpha-2-HS-glycoprotein	P02765	Alpha-2-HS-g
9	Alpha-2-macroglobulin	P01023	Alpha-2-macrogl
10	Antithrombin-III	P01008	Antithrombin-III
11	Apolipoprotein A-II	P02652	ApolipoA-II
12	Apolipoprotein A-IV	P06727	ApolipA-IV
13	Apolipoprotein B-100	P04114	ApolipoB-100
14	Apolipoprotein C-I	P02654	ApolipC-I
15	Apolipoprotein C-II	P02655	ApolipC-II
16	Apolipoprotein C-III	P02656	ApolipoC-III
17	Apolipoprotein M	O95445	ApolipM
18	Apolipoprotein(a)	P08519	Apolipo(a)
19	Beta-Ala-His dipeptidase	Q96KN2	Beta-Ala-His_dip
20	Carbonic anhydrase 1	P00915	Carbonic anh1
21	Carboxypeptidase B2	Q96IY4	Carboxypept_B2
22	Cholinesterase	P06276	Cholinesterase
23	Coagulation factor IX	P00740	Coag_fact_IX
24	Coagulation factor V	P12259	Coag_fact_V
25	Coagulation factor X	P00742	Coag_fact_X
26	Complement C1q subcomponent subunit C	P02747	Compl_C1q subc-s_C
27	Complement C1r subcomponent	P00736	Compl_C1r_subc

No.	Protein	Uniprot Accession Number	Abbreviated name
28	Complement C1s subcomponent	P09871	Compl_ C1s_subc
29	Complement C3	P01024	Compl_C3
30	Complement C5	P01031	Compl_C5
31	Complement component C7	P10643	Compl_comp_C7
32	Complement component C9	P02748	Compl_ct C9
33	Complement factor B	P00751	Compl fB
34	Complement factor I	P05156	Compl__I
35	Corticosteroid-binding globulin	P08185	Cortic-bgl
36	Cystatin-C	P01034	Cystatin-C
37	Endothelial protein C receptor	Q9UNN8	Endo_protC_rec
38	Fetuin-B	Q9UGM5	Fetuin-B
39	Fibrinogen beta chain	P02675	Fibrinogen_b-chain
40	Fibronectin	P02751	Fibronectin
41	Fibulin-1	P23142	Fibulin-1
42	Galectin-3-binding protein	Q08380	Galectin-3-bindingP
43	Gelsolin	P06396	Gelsolin
44	Glutathione peroxidase 3	P22352	Glutathione_POX_3
45	Haptoglobin	P00738	Haptoglobin
46	Hemopexin	P02790	Hemopexin
47	Heparin cofactor 2	P05546	Heparin_cof2
48	Hepatocyte growth factor-like protein	P26927	Hepat_GF-likeP
49	Hyaluronan-binding protein 2	Q14520	Hyaluronan-bp2
50	Intercellular adhesion molecule 1	P05362	Interc_adh_mol_1
51	Kallistatin	P29622	Kallistatin
52	Keratin-type II cytoskeletal 2 epidermal	P35908	Keratin-II_cyt2epid
53	Kininogen-1	P01042	Kininogen-1
54	Leucine-rich alpha-2-glycoprotein	P02750	Leu_rich_a2-glyc
55	Lipopolysaccharide-binding protein	P18428	Lipopol_BP
56	L-selectin	P14151	L-selectin
57	Lysozyme C	P61626	Lysozyme_C
58	Mannan-binding lectin serine protease 1	P48740	Mannan-b_lec_ser_prot_1

No.	Protein	Uniprot Accession Number	Abbreviated name
59	Mannan-binding lectin serine protease 2	O00187	Mannan-b_e_s_p2
60	Peroxiredoxin-2	P32119	Peroxiredoxin-2
61	Phosphatidylinositol-glycan-specific phospholipase D	P80108	PI-glycan-sp_phlip_D
62	Phospholipid transfer protein	P55058	Phospholipid_tp
63	Pigment epithelium-derived factor	P36955	Pigment epith_DRF
64	Plasma protease C1 inhibitor	P05155	P-protC1_inh
65	Plasminogen	P00747	Plasminogen
66	Protein S100-A9	P06702	Protei_S100-A9
67	Protein Z-dependent protease inhibitor	Q9UK55	Protein Z-dep_p_inh
68	Prothrombin	P00734	Prothrombin
69	Retinol-binding protein 4	P02753	Retinol-bp4
70	Serotransferrin	P02787	Serotransferrin
71	Serum paraoxonase/arylesterase 1	P27169	S-paraoxo_aryl_ase1
72	SPARC	P09486	SPARC
73	Tenascin-X | Putative tenascin-XA	P22105	Tenascin-X
74	Thrombospondin-1	P07996	Thrombosp-1
75	Transthyretin	P02766	Transthyretin
76	Vasorin	Q6EMK4	Vasorin
77	Vitamin K-dependent protein S	P07225	VitK-dep_ p_S
78	Vitamin K-dependent protein Z	P22891	VitK-dep_pZ
79	Vitronectin	P04004	Vitronectin
80	Zinc-alpha-2-glycoprotein	P25311	Zinc-a2-glyc
81	78 kDa glucose-regulated protein	P11021	78 kDa gluc_reg_prot

Table S5. Gene Ontology (GO) analysis related to cellular component.

#term ID	term description	observed gene count	background gene count	strength	false discovery rate	matching proteins in the network (IDs)	matching proteins in the network (labels)
GO:0072562	Blood microparticle	6	115	19	0.0000138	9606.ENSP00000226218,9606.ENSP00000226355, 9606.ENSP00000265023,9606.ENSP00000323929, 9606.ENSP00000350425,9606.ENSP00000377783	VTN,AFM,KNG1,A2M,APOA4,PROS1
GO:0070062	Extracellular exosome	11	2099	9	0.00012	9606.ENSP00000226218,9606.ENSP00000226355, 9606.ENSP00000227667,9606.ENSP00000261267, 9606.ENSP00000265023,9606.ENSP00000323929, 9606.ENSP00000350425,9606.ENSP00000360522, 9606.ENSP00000377783,9606.ENSP00000381448, 9606.ENSP00000383690	VTN,AFM,APOC3,LYZ,KNG1,A2M,APOA4,RBP4,PROS1,CST3,MASP2
GO:0062023	Collagen-containing extracellular matrix	6	396	136	0.00373	9606.ENSP00000226218,9606.ENSP00000227667, 9606.ENSP00000265023,9606.ENSP00000323929, 9606.ENSP00000350425,9606.ENSP00000381448	VTN,APOC3,KNG1,A2M,APOA4,CST3
GO:0031093	Platelet alpha granule lumen	3	68	182	22	9606.ENSP00000265023,9606.ENSP00000323929, 9606.ENSP00000377783	KNG1,A2M,PROS1
GO:0005788	Endoplasmic reticulum lumen	4	308	129	57	9606.ENSP00000226218,9606.ENSP00000265023, 9606.ENSP00000350425,9606.ENSP00000381448	VTN,KNG1,APOA4,CST3
GO:0042627	Chylomicron	2	13	236	57	9606.ENSP00000227667,9606.ENSP00000350425	APOC3,APOA4
GO:0034774	Secretory granule lumen	4	324	127	60	9606.ENSP00000261267,9606.ENSP00000265023, 9606.ENSP00000323929,9606.ENSP00000377783	LYZ,KNG1,A2M,PROS1
GO:0034361	Very-low-density lipoprotein particle	2	20	218	93	9606.ENSP00000227667,9606.ENSP00000350425	APOC3,APOA4
GO:0030141	Secretory granule	5	845	95	131	9606.ENSP00000261267,9606.ENSP00000265023, 9606.ENSP00000323929,9606.ENSP00000377783, 9606.ENSP00000381448	LYZ,KNG1,A2M,PROS1,CST3
GO:0034364	High-density lipoprotein particle	2	29	202	159	9606.ENSP00000227667,9606.ENSP00000350425	APOC3,APOA4
GO:0031410	Cytoplasmic vesicle	7	2386	64	253	9606.ENSP00000227667,9606.ENSP00000261267, 9606.ENSP00000265023,9606.ENSP00000323929, 9606.ENSP00000350425,9606.ENSP00000377783, 9606.ENSP00000381448	APOC3,LYZ,KNG1,A2M,APOA4,PROS1,CST3
GO:1904724	Tertiary granule lumen	2	55	174	400	9606.ENSP00000261267,9606.ENSP00000381448	LYZ,CST3

Table S6. GO analysis related to the biological process revealing the enrichment of GO terms related to chylomicron remodeling and assembly.

#term ID	term description	observed gene count	background gene	strength	false discovery rate	matching proteins in your network (IDs)	matching proteins in your network (labels)																		
GO:0034371	Chylomicron remodeling	2	9	2.52	0.0164	9606.ENSP00000227667	9606.ENSP00000350425	APOC3	APOA4																
GO:0034378	Chylomicron assembly	2	10	2.48	0.0164	9606.ENSP00000227667	9606.ENSP00000350425	APOC3	APOA4																
GO:0033700	Phospholipid efflux	2	12	2.4	0.0215	9606.ENSP00000227667	9606.ENSP00000350425	APOC3	APOA4																
GO:0010896	Regulation of triglyceride catabolic process	2	14	2.33	0.0250	9606.ENSP00000227667	9606.ENSP00000350425	APOC3	APOA4																
GO:0007597	Blood coagulation	intrinsic pathway	2	17	2.25	0.0287	9606.ENSP00000265023	9606.ENSP00000323929	KNG1	A2M															
GO:0043691	Reverse cholesterol transport	2	17	2.25	0.0287	9606.ENSP00000227667	9606.ENSP00000350425	APOC3	APOA4																
GO:0034375	High-density lipoprotein particle remodeling	2	18	2.22	0.0290	9606.ENSP00000227667	9606.ENSP00000350425	APOC3	APOA4																
GO:0051004	Regulation of lipoprotein lipase activity	2	22	2.14	0.0350	9606.ENSP00000227667	9606.ENSP00000350425	APOC3	APOA4																
GO:0033344	Cholesterol efflux	2	23	2.12	0.0362	9606.ENSP00000227667	9606.ENSP00000350425	APOC3	APOA4																
GO:0030195	Negative regulation of blood coagulation	3	47	1.98	0.0113	9606.ENSP00000226218	9606.ENSP00000265023	9606.ENSP00000377783	VTN	KNG1	PROS1														
GO:0030449	Regulation of complement activation	3	56	1.91	0.0113	9606.ENSP00000226218	9606.ENSP00000323929	9606.ENSP00000377783	VTN	A2M	PROS1														
GO:0001523	Retinoid metabolic process	3	108	1.62	0.0250	9606.ENSP00000227667	9606.ENSP00000350425	9606.ENSP00000360522	APOC3	APOA4	RBP4														
GO:0002576	Platelet degranulation	3	129	1.54	0.0291	9606.ENSP00000265023	9606.ENSP00000323929	9606.ENSP00000377783	KNG1	A2M	PROS1														
GO:0015850	Organic hydroxy compound transport	3	145	1.49	0.0350	9606.ENSP00000227667	9606.ENSP00000350425	9606.ENSP00000360522	APOC3	APOA4	RBP4														
GO:0032102	Negative regulation of response to external stimu	4	367	1.21	0.0291	9606.ENSP00000226218	9606.ENSP00000265023	9606.ENSP00000323929	9606.ENSP00000377783	VTN	KNG1	A2M	PROS1												
GO:0002697	Regulation of immune effector process	4	418	1.16	0.0381	9606.ENSP00000226218	9606.ENSP00000323929	9606.ENSP00000360522	9606.ENSP00000377783	VTN	A2M	RBP4	PROS1												
GO:0010951	Negative regulation of endopeptidase activity	5	248	1.48	0.0030	9606.ENSP00000226218	9606.ENSP00000265023	9606.ENSP00000323929	9606.ENSP00000377783	9606.ENSP00000381448	VTN	KNG1	A2M	PROS1	CST3										
GO:0045055	Regulated exocytosis	5	697	1.03	0.0260	9606.ENSP00000261267	9606.ENSP00000265023	9606.ENSP00000323929	9606.ENSP00000377783	9606.ENSP00000381448	LYZ	KNG1	A2M	PROS1	CST3										
GO:0051346	Negative regulation of hydrolase activity	6	450	1.3	0.0030	9606.ENSP00000226218	9606.ENSP00000227667	9606.ENSP00000265023	9606.ENSP00000323929	9606.ENSP00000377783	9606.ENSP00000381448	VTN	APOC3	KNG1	A2M	PROS1	CST3								
GO:0051241	Negative regulation of multicellular organismal pr	7	1231	0.93	0.0113	9606.ENSP00000226218	9606.ENSP00000227667	9606.ENSP00000265023	9606.ENSP00000350425	9606.ENSP00000360522	9606.ENSP00000377783	9606.ENSP00000381448	VTN	APOC3	KNG1	APOA4	RBP4	PROS1	CST3						
GO:0051336	Regulation of hydrolase activity	7	1284	0.91	0.0113	9606.ENSP00000226218	9606.ENSP00000227667	9606.ENSP00000265023	9606.ENSP00000323929	9606.ENSP00000350425	9606.ENSP00000377783	9606.ENSP00000381448	VTN	APOC3	KNG1	A2M	APOA4	PROS1	CST3						
GO:0006810	Transport	10	4353	0.54	0.0238	9606.ENSP00000226218	9606.ENSP00000226355	9606.ENSP00000227667	9606.ENSP00000261267	9606.ENSP00000265023	9606.ENSP00000323929	9606.ENSP00000350425	9606.ENSP00000360522	9606.ENSP00000377783	9606.ENSP00000381448	VTN	AFM	APOC3	LYZ	KNG1	A2M	APOA4	RBP4	PROS1	CST3

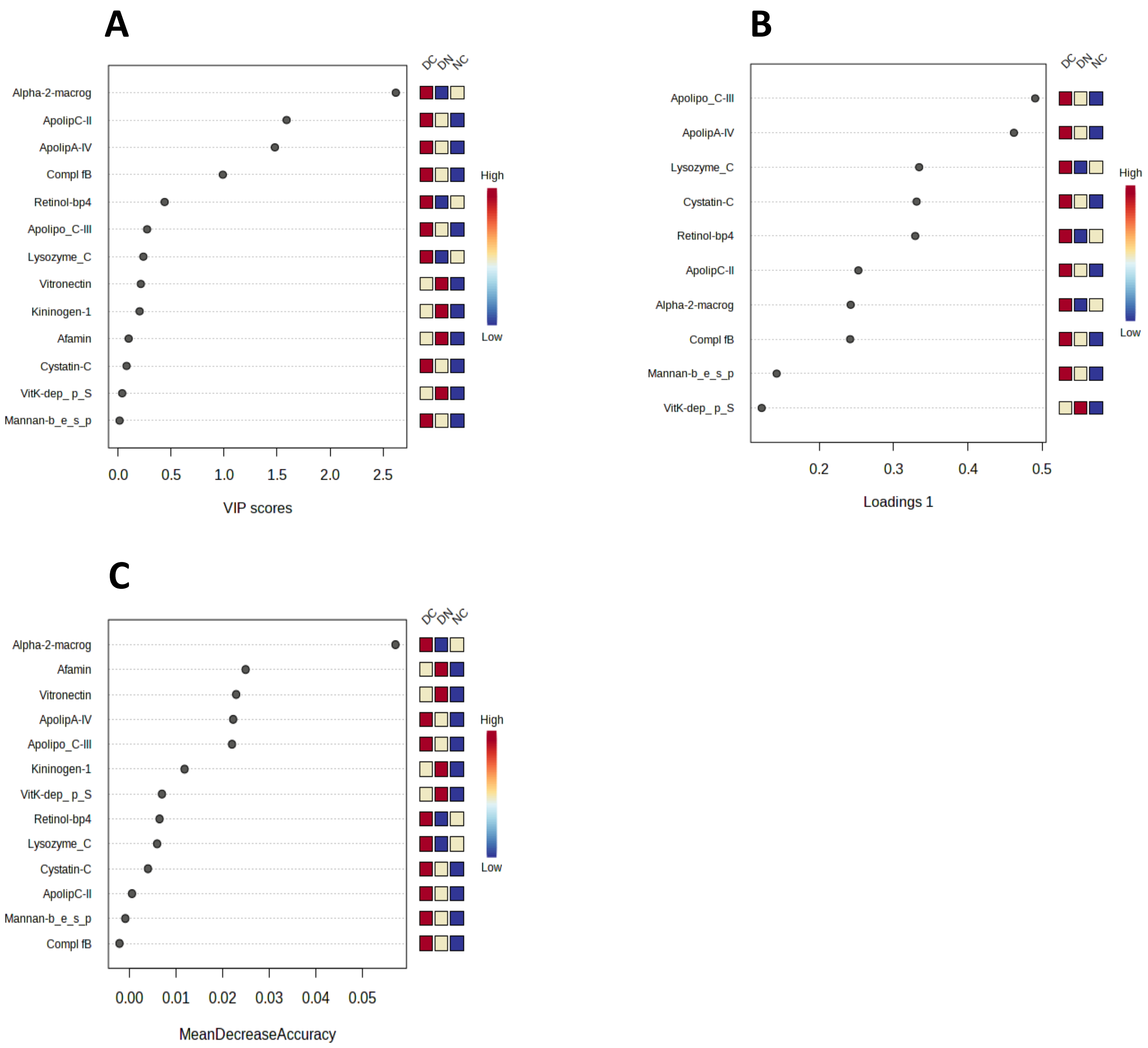

Figure S1. Validation of the 13 proteins selected with ANOVA. **Panel A:** Plot showing Important features identified by **PLS-DA**. The colored boxes on the right indicate the relative concentrations of the corresponding metabolite in each group under study. Variable Importance in Projection (VIP) is a weighted sum of squares of the PLS loadings taking into account the amount of explained variation in each dimension. **Panel B**: Plot showing the variables selected by the **sPLS-DA** model for a given component. The variables are ranked by the absolute values of their loadings. **Panel C**: Significant features identified by Random Forest. The features are ranked by the mean decrease in classification accuracy when they are permuted.

Table S7. Summary of the features of the 13 proteins identified by ANOVA, according to the different non-parametric employed statistical procedures. The relevance of the proteins is indicated with semi-quantitative approximate scoring.

**Features identified with ANOVA**	**Uniprot Accession Number**	**Validation of relevant features by**		
		**PLS-DA**	**sPLS-DA**	**Random Forest**
Apolipoprotein A-IV	P06727	+++	+++	++±
Apolipoprotein C-III	P02656	+	+++	++±
Afamin	P43652	+	-	++±
Vitamin K-dependent protein S	P07225	±	±	+
Lysozyme C	P61626	+	++±	+
Kininogen-1	P01042	+	-	+±
Vitronectin	P04004	+	-	++±
Retinol-binding protein 4	P02753	+±	++±	+
Cystatin-C	P01034	±	++±	+
Alpha-2-macroglobulin	P01023	+++	++	+++
Apolipoprotein C-II	P02655	+++	++	±
Complement factor B	P00751	++	++	±
Mannan-binding lectin serine protease 2	P01034	±	+	±

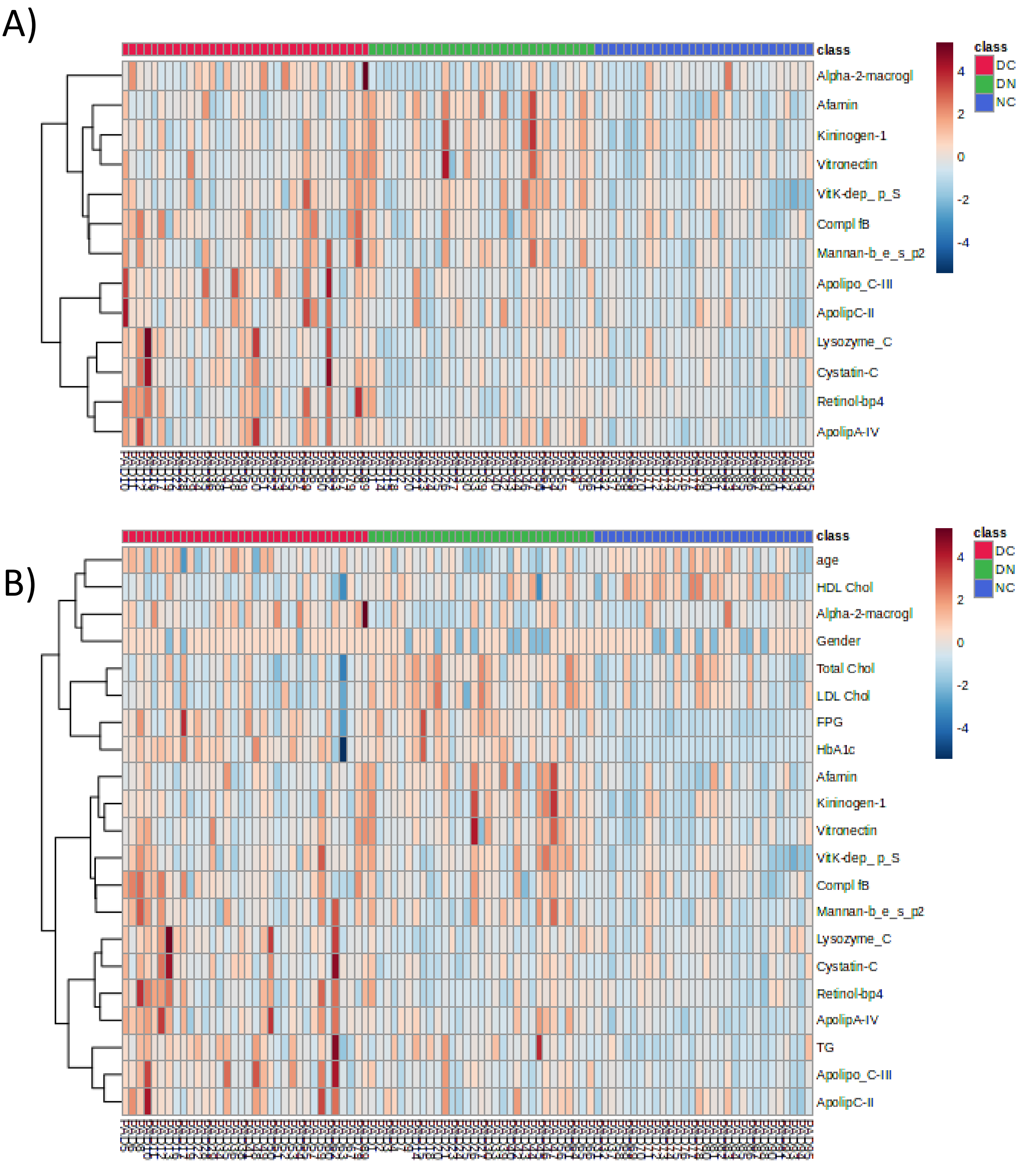

Figure S2. A) Clustering result shown as heatmap (distance measure using Euclidean, and clustering algorithm using Ward method), obtained on the 13 selected proteins significant at ANOVA; differently from Figure 5, no ordering of groups was here applied, to show the overall pattern of protein distribution among groups. Heatmap color intensity is proportional to the parameter's value (blue to red), as in the reported scale on the right of the figure. B) Clustering result including also factors such as age, gender, lipid profile, and glucose parameters.

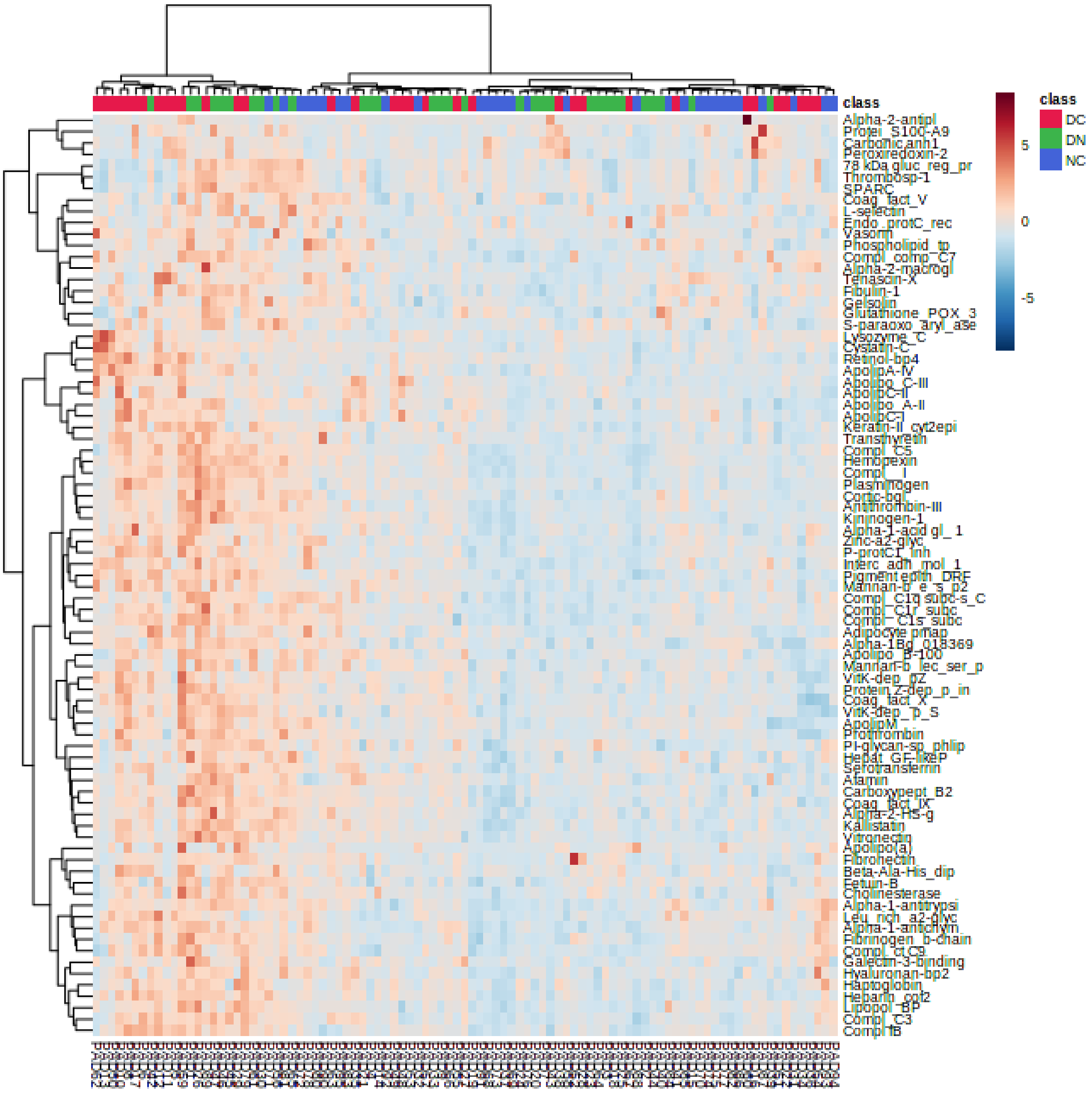

Figure S3. Clustering result shown as heatmap (distance measure using Euclidean, and clustering algorithm using Ward method), obtained on the overall panel of 81 proteins.

Table S8. Effect summary of nominal logistic plot for three paired comparisons between groups of subjects by using the 13 selected proteins. Logworth and *p* value for each item are presented. Whole model test was successfully accomplished in any cases with overall significance of *p*<0.0001.

Comparison NC vs DN

Source	Logworth	PValue
Compl fB	2.259	0.00551
Afamin	1.523	0.03002
ApolipA-IV	1.320	0.04790
VitK-dep_ p_S	1.311	0.04882
Cystatin-C	1.263	0.05455
Vitronectin	0.726	0.18777
Alpha-2-macrogl	0.685	0.20632
Retinol-bp4	0.631	0.23366
ApolipC-II	0.467	0.34155
Mannan-b_e_s_p2	0.405	0.39381
Kininogen-1	0.210	0.61694
Lysozyme_C	0.196	0.63717
Apolipo_C-III	0.156	0.69757

Comparison DC vs DN

Source	Logworth	PValue
Apolipo_C-III	31.113	0.00000
Kininogen-1	23.590	0.00000
Alpha-2-macrogl	9.960	0.00000
Cystatin-C	9.182	0.00000
Compl fB	4.809	0.00002
Afamin	0.065	0.86188
Mannan-b_e_s_p2	0.011	0.97523
ApolipA-IV	0.001	0.99873
Lysozyme_C	0.000	0.99923
VitK-dep_ p_S	0.000	0.99990
Retinol-bp4	0.000	0.99998
ApolipC-II	0.000	1.00000
Vitronectin	0.000	1.00000

Comparison DC vs NC

Source	Logworth	PValue
Kininogen-1	2.914	0.00122
Apolipo_C-III	2.439	0.00364
VitK-dep_ p_S	1.640	0.02291
Vitronectin	1.563	0.02732
ApolipA-IV	1.416	0.03839
Alpha-2-macrogl	1.394	0.04040
Afamin	0.971	0.10698
Mannan-b_e_s_p2	0.606	0.24790
Lysozyme_C	0.530	0.29490
Cystatin-C	0.424	0.37630
Retinol-bp4	0.261	0.54852
ApolipC-II	0.216	0.60804
Compl fB	0.097	0.79898
